# Detection superiority of 7 T MRI protocol in patients with epilepsy and suspected focal cortical dysplasia

**DOI:** 10.1007/s13760-016-0662-x

**Published:** 2016-07-08

**Authors:** A. J. Colon, M. J. P. van Osch, M. Buijs, J. v. d. Grond, P. Boon, M. A. van Buchem, P. A. M. Hofman

**Affiliations:** 1 Academic Center for Epileptology Kempenhaeghe, Maastricht Universitair Medisch Centrum + (MUMC+), Sterkselseweg 65, 5590 VE Heeze, The Netherlands; 2 Department of Radiology, Leiden University Medical Centre, Leiden, The Netherlands; 3 Department of Neurology, University Hospital Gent, Ghent, Belgium

**Keywords:** 7 T MRI, Focal cortical dysplasia, Epilepsy, Human, Misdiagnosis

## Abstract

In 11 adult patients with suspicion of Focal cortical dysplasia (FCD) on 1.5 T (*n* = 1) or 3 T (*n* = 10) magnetic resonance imaging (MRI), 7 T MRI was performed. Visibility, extent, morphological features and delineation were independently rated and subsequently discussed by three observers. Additionally, head-to-head comparisons with corresponding 3 T images were made in the eight patients with a previous 3 T MRI and sustained suspicion of FCD. Comparison with histopathology was done in the five patients that underwent surgery. All lesions, seen at 1.5 and 3 T, were also recognized on 7 T. At 7 T FLAIR highlighted the FCD-like lesions best, whereas T2 and T2* were deemed better suited to review structure and extent of the lesion. Image quality with the used 7 T MRI setup was higher than the quality with the used 3 T MRI setup. In 2 out of 11 patients diagnosis changed, in one after re-evaluation of the images, and in the other based on histopathology. With the used 7 T MRI setup, FCD-like lesions can be detected with more confidence and detail as compared to lower field strength. However, concordance between radiologic diagnosis and final diagnosis seems to be lower than expected.

**Electronic supplementary material:**

The online version of this article (doi:10.1007/s13760-016-0662-x) contains supplementary material, which is available to authorized users.

## Introduction

20–40 % of epilepsy patients are drug resistant. In this group resective surgery, if possible, is the treatment of choice [1, 2]. Finding a lesion on magnetic resonance imaging (MRI) is of clinical importance, as presence of a lesion is associated with a higher chance of developing drug resistant (refractory) epilepsy [3] and increased success rate of surgery [4–6]. Sensitivity of MRI for brain lesions partly depends on the system’s magnetic field strength [7–12]. Higher field strength gives rise to higher signal-to-noise ratio, which allows for higher anatomical resolution and increased sensitivity for contrast mechanisms such as those based on iron [13–18]. Most studies in epilepsy patients have been performed using 1.5 and 3 T MRI systems. MRI systems operating at a magnetic field strength of 7 T may have added value for epilepsy patients [19] especially because they may have a higher sensitivity for focal cortical dysplasia (FCD) and decrease the number of MRI-occult FCDs. In surgical series, FCD is a common pathologic finding with a reported presence in 8 % [20] to 53 % of the operated epileptic patients [21]. 20–30 % of patients with postsurgical proven FCD were MRI-negative [3, 22, 23]. No systematic comparison between 3 and 7 T MRI appearance of FCD is available. On the other hand, MRI diagnosis of FCD can be erroneous. FCD’s can be hard to distinguish from gliomas, with a preference for FCD on frontal locations or a lesser distinct high intensity on T2 weighted images than in case of glioma [24].

The most frequently described MRI features of FCD include: increased cortical thickness, blurred grey/white matter junction, increased signal on T2, decreased signal on T1 of the subcortical white matter and gyration anomalies [22, 23]. The most typical feature highly specific for FCD type II is alteration of white matter signal towards the ventricle, the “transmantle sign” [25]. Presence of a focal lesion (e.g., mesiotemporal sclerosis) combined with FCD defines FCD type III. FCD can be characterised by combinations of several of the above mentioned MRI features [26]. In many patients only a subset of these MRI features are detected [26]. As presentation of an abnormality can be dependent on the field strength, we wanted to study the presentation on 7 T MRI of FCD’s previously described on lower field strength MRI. Further more findings on 3 T MRI if available were compared to the appearance at 7 T MRI and radiologic diagnosis was compared to histopathology in operated cases.

## Methods

Ten adult patients, diagnosed with localisation related epilepsy and presence of a lesion diagnosed as FCD on 3 T (*n* = 10) or 1.5 T (*n* = 1) MRI, were included (patient characteristics: Table 1). An additional patient (patient 5) was excluded from further analysis as due to technical failure on 7 T MRI the signal-to-noise ratio was too low. All lesions were located extra-temporal, one with temporal extension and one with dual pathology. Standard MRI exclusion criteria were applied. Presence of a dental retainer wire was added as a 7 T-specific MRI exclusion criterion. At present this is not an exclusion criterion any more [27]. Informed consent was obtained from all individual participants included in the study. The study was approved by the Institutional Review Board of LUMC (Leiden Universitair Medisch Centrum).

**Table 1:** Patient characteristics of patients with FCD-like lesion on prior MRI

Patient	Age	Sex	Location of lesion on previous MRI	Semiology
1	34	M	Right frontal	Conscious, forced head version to right followed by secondary generalisation
2	22	M	Left parietal	Short lasting: light headedness, goosebumps, staring, incorrect answers, bilateral manual automatisms
3	25	V	Left frontal	Stretching right arm and inability to speak
4	44	M	Left temporo-occipital	Lowered consciousness, automatisms, wandering
5	47	M	Left parietal	Nightly symmetric or asymmetric tonic contractions or very brief myoclonias
6	21	M	Right parietal	Visual hallucinations, lowered consciousness, hypermotor behaviour
7	47	V	Right hand knob	Pounding sensation left thumb, painfull contraction left hand
8	20	M	Left frontal (ganglioglioma) and left parietal (FCD)	Sensation of mouth movement, inability to speak, problems with co-ordination. If secondary generalisation then post-ictal visual disturbances
9	34	M	Left parietal	Vibrating sensation right face, sensation of falling to the right (actually going to the left), raising right arm, staring. Fully conscious
10	36	V	Left parietal	1. Sensation of jaw cramp, tingeling gums, hypersalivation, aphasia 2. Short epigastric aura, tonic contraction right arm, secondary generalisation
11	43	V	Right frontal	1. Cephalic sensation, fear, sensation of short of breath 2. During sleep head turning to left, orofacial automatisms and/or bipedaling and/or hypertonia left arm

7 T MRI was performed on a Philips Achieva platform (Philips Healthcare, Cleveland, Ohio) using a 32 channel receive head coil with quadrature transmit. The following sequences were used: 3D T1 (TR 4.2 ms, TE 1.88 ms, voxel-size 0.9 × 0.9 × 0.9 mm), 3D FLAIR (TR 7900 ms, TE 300 ms, TI 2200 ms, voxel-size 0.85 × 0.85 × 0.85 mm), T2 TSE (TR 3000 ms, TE 58 ms, voxel-size 0.5 × 0.5 × 1 mm) and T2* (TR 1764 ms, TE 25 ms, voxel-size 0.24 × 0.24 × 1 mm). Total acquisition time was under 1 h, which was considered acceptable for possible future use in clinical practice. The 3 T MRI images were acquired using a 16 channel receive head coil and a state-of-the-art epilepsy protocol [3D-T1 (TR 8.1 ms, TE 3.7 ms, voxel 1 × 1 × 1 mm), T2 (TR 3000 ms, TE 80 ms, voxel 0.5 × 0.5 × 5 mm), T2* in the last 5 patients (TR 777 ms, TE 16 ms, voxel 0.9x1.1x5 mm), IR (TR 120 ms, TE 10 ms, TI 400 ms, voxel 0.4 × 0.6 × 2 mm), FLAIR (TR 8000 ms, TE 50 ms, TI 2400 ms, voxel 1.1 × 1.1 × 0.5 mm)] performed on a Philips 3.0 T Achieva platform (Philips Medical systems, Best, The Netherlands). Diagnosis of FCD was made by an experienced neuroradiologist in a tertiary epilepsy centre (PH). In one patient only 1.5 T MRI, made in a referring hospital, was available; since this 1.5 T MRI examination showed clearly an FCD, additional 3 T MRI was deemed unnecessary.

Patient charts were examined by two neurologists (AC, LW), with experience in analysing patients for epilepsy surgery. Based on medical history, semiology, EEG, and if available seizure-recordings clinical estimation of the location of the epileptogenic focus was formulated. If the patient underwent surgery data on histopathology were noted. Data were compared to the MRI results.

Two experienced neuroradiologists (MvB, PH) and a neurologist (AC) visually inspected the images.

All observers were aware of the presence of an MRI-detectable lesion. Windowing was individually adapted to gain optimal contrast. Orientation of slides with the highest visibility of the abnormality was chosen separately for each field strength. Presence and characteristics of a possible FCD (see Table 2) were noted using a predefined scoring system. Seven features were scored: blurring of the grey–white matter junction, focal thickening of the cortex, focal increased intensity, presence of a transmantle sign, clear demarcation of transition to normal cortex, gyral pattern and abnormal internal structure. A flag-like appearance of the FCD was noted in several patients. This characteristic was added to the study.

**Table 2:** Features of FCD recognized on 7 T MRI, all sequences combined

Patient	2	3	4	6	7	8	9	10	11	Overall
Blurring	+	+	+	+	+	+	+	+	+	9/9
Focal thickening	+	+	+	+	−	+	+	+	+	8/9
Focal increased intensity	+	+	+	+	+	+	+	+	+	9/9
Transmantle sign	−	+	−	−	+	+	+	+	+	6/9
Transition to normal cortex	v	s	s	s	v	s	s	s	s	2 vague, 7 sharp
Gyral pattern	norm	abn	abn	abn	norm	norm	abn	abn	abn	6/9
Abnormal internal structure	+	+	+	+	+	+	+	+	+	9/9
Total	4/7	7/7	6/7	6/7	4/7	6/7	7/7	7/7	7/7	

+ present, − not visible, *v* vague, *s* scharp, *norm* normal, *abn* abnormal

In eight patients with a 3 T MRI and a sustained pre-operative diagnosis of FCD the features of the FCD on T2 and FLAIR images were rated for visibility using a Likert scale from 1 to 3 [28]: 1 indicating 7 T superior to 3 T, 2 equal quality, and 3 indicating 3 T superior to 7 T. Comparison was made between the same sequences using a Sign test (http://www.fon.hum.uva.nl/Service/Statistics/Sign_Test.html). Non-difference was set as the null hypothesis. As T2* was not available in all 3 T MRIs no comparison between T2* on 7 T and on 3 T sequence was made.

## Results

In none of the patients abnormalities were found on 7 T MRI that were not observed at lower field strength and all lesions visible at lower field strength were visible at 7 T. In the ten analysed patients, both neurologists agreed that the stereotyped seizures were based on a single epileptogenic focus. In each patient one or more hypotheses on the possible locations of this epileptogenic focus could be postulated. Comparison between these hypotheses and the location of the lesion on MRI showed concordance in each individual patient.

By visual inspection in all patients the lesion was detected on 7 T MRI without prior knowledge of the location as seen at lower field strength. In patient 1 the diagnosis changed from FCD to cavernoma based on the 7 T MRI and re-evaluation of the 3 T images. The treating physician was informed. Further visual analysis of the 7 T MRI was done for the remaining nine patients.

**Fig. 1:**
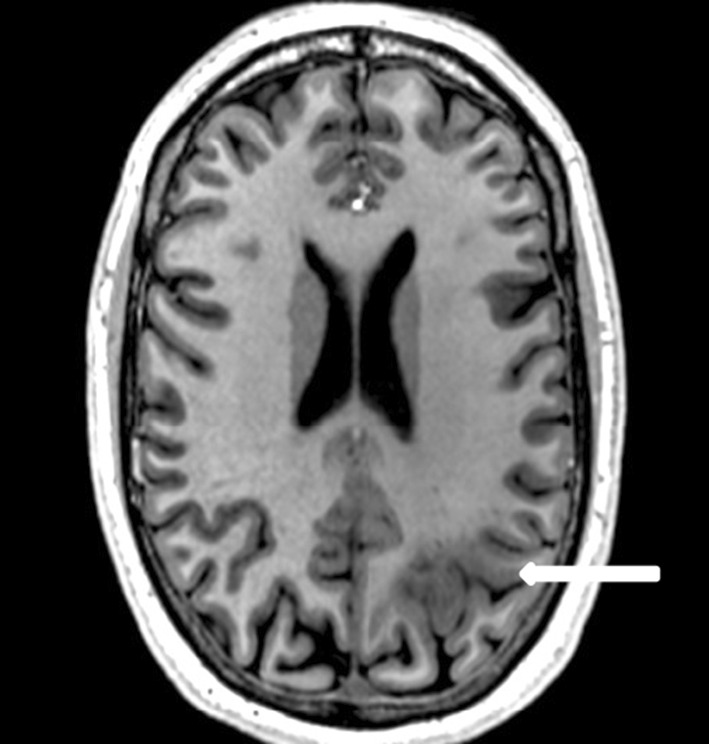
7 T T1 image showing cortical thickening and blurring

**Fig. 2:**
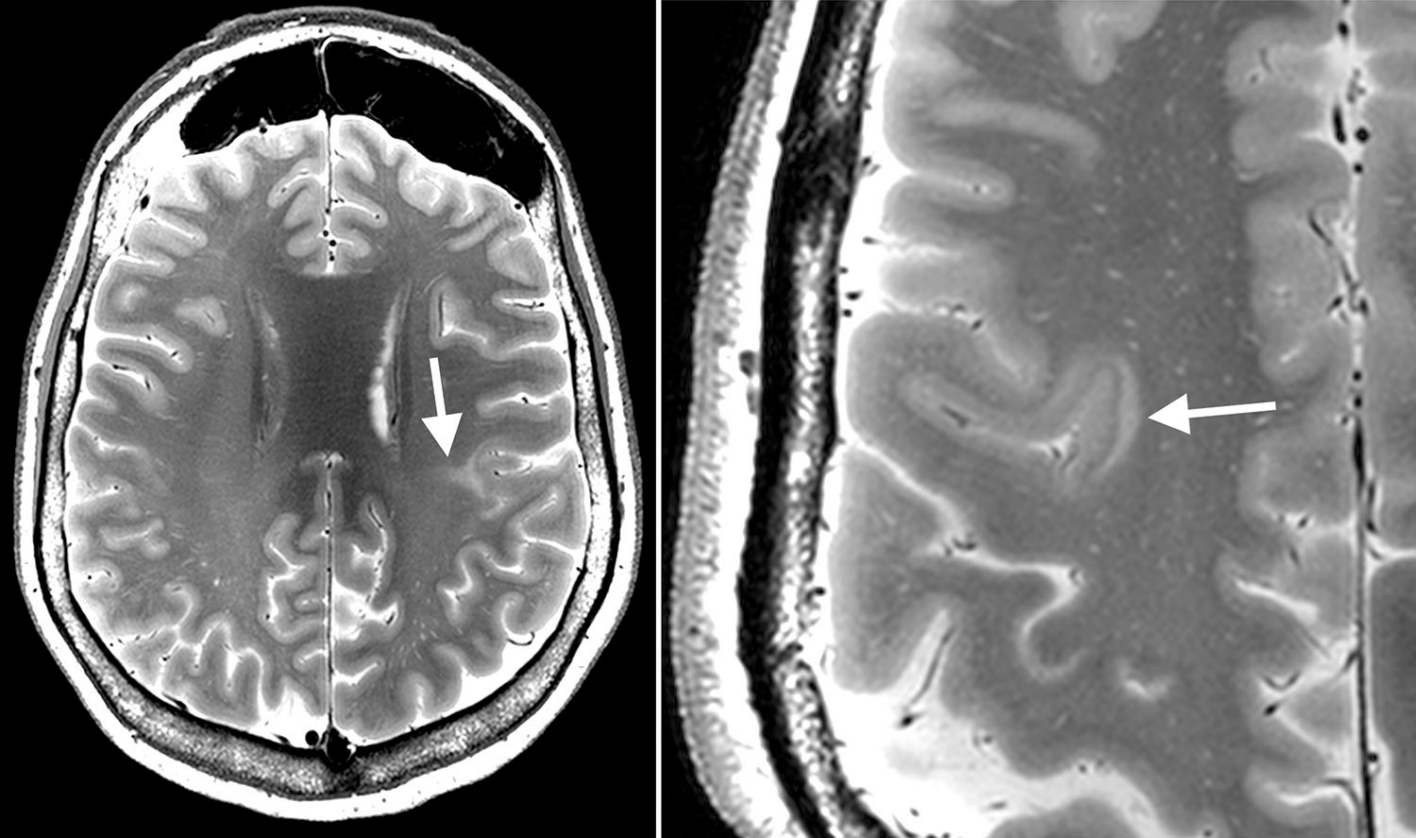
7 T T2 image showing cortical thickning, transmantle sign (*left*) and flag-like appearence at the *bottom* of the sulcus in FCD (*right* more pronounced than *left*)

**Fig. 3:**
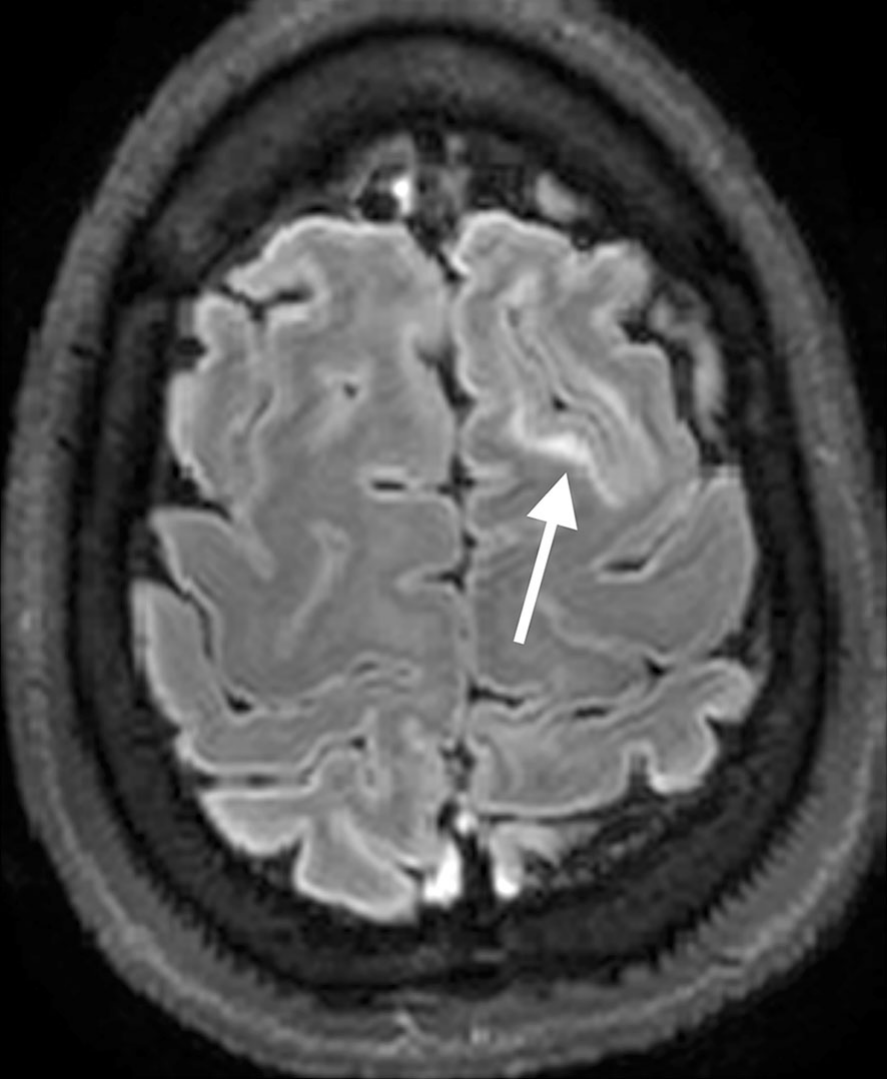
7 T FLAIR image, showing highlighted FCD

**Fig. 4:**
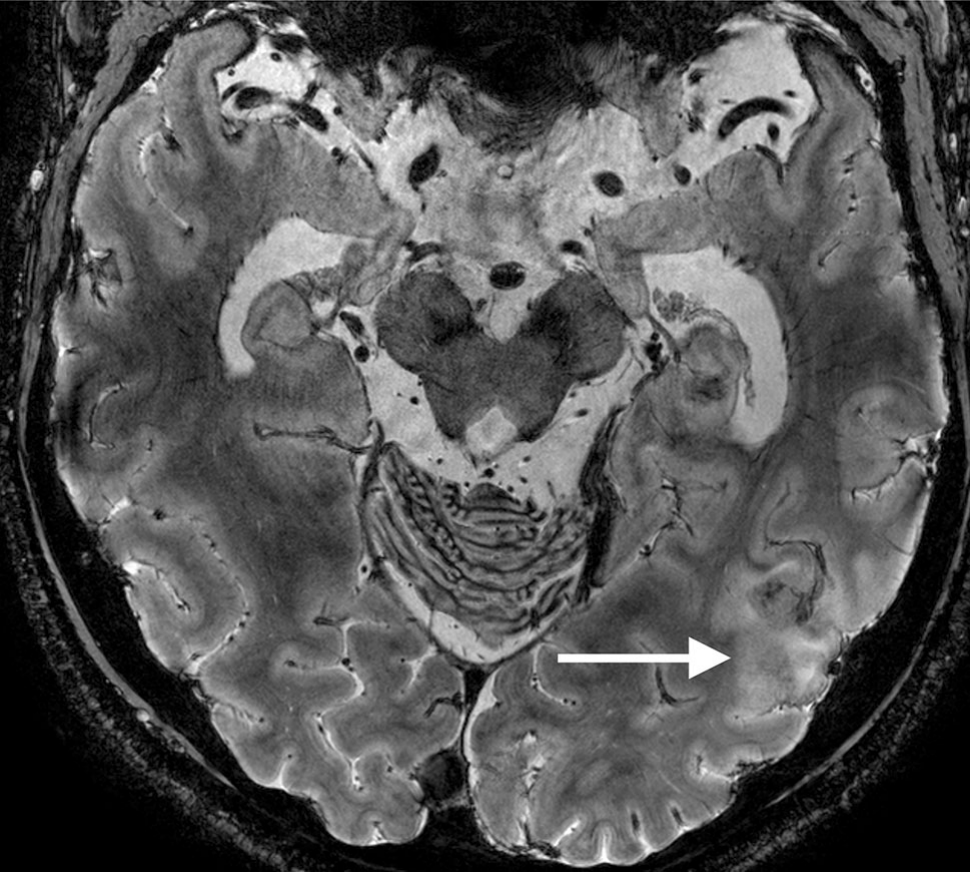
7 T T2* image showing abnormal internal structure of FCD

On the T1-weighted images cortical thickening and blurring were most prominent (Fig. 1). A hypo-intense line at the grey–white matter junction was observed on T2 weighted images (Fig. 2) in eight patients creating a typical three layer flag-like appearance. Detection of the FCD was readily made on the FLAIR images (Fig. 3), whereas the abnormal internal structure of FCDs was most clearly seen on T2* (Fig. 4). Supplementary online data shows more examples. Of the seven imaging features, four (blurring of grey–white matter, focal signal increase, visibility of transition to normal cortex, abnormal internal structure) were observed in all nine patients. Focal thickening was observed in eight patients, funnel shaped extension in and the presence of an abnormal gyral pattern in six patients (Table 2). The flag-like appearance was noted in all but patient 3.

**Fig. 5:**
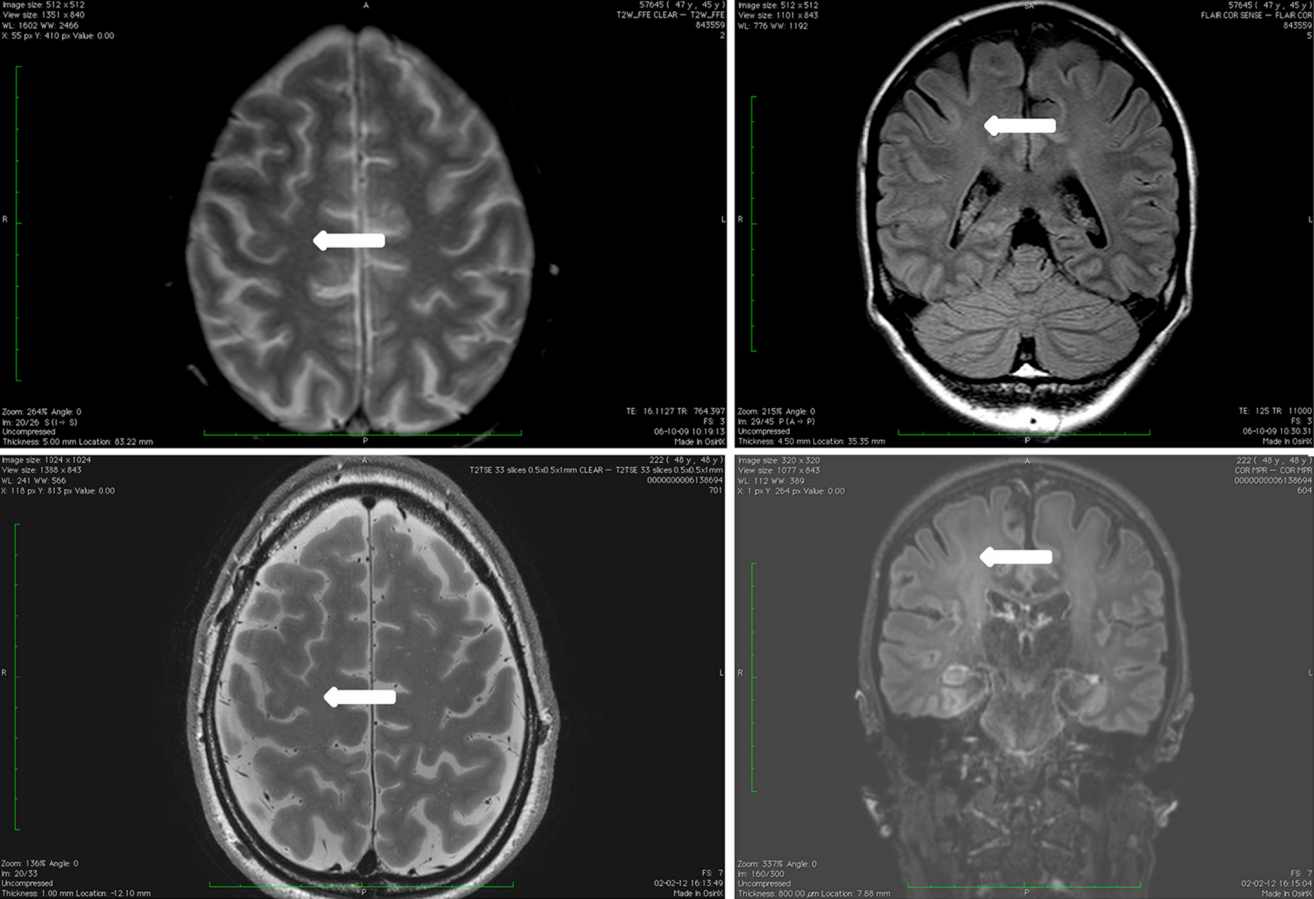
Comparison between 3 (*top*) and 7 (*bottom*) T images of a patient with known FCD *right hand* knob to deep-of-the-sulcus. From *left* to *right* T2, FLAIR

In the eight patients in whom 3 T MRI was available, 7 T MRI depiction scored significantly better than 3 T MRI for blurring (*p* < 0.01), abnormalities of internal structure (*p* < 0.01) and demarcation of transition to normal cortex (*p* < 0.02) (Fig. 5) on T2 and for abnormalities of internal structure (*p* < 0.04) on FLAIR. Although not statistically significant, 7 T MRI tended to be superior to 3 T on another 7 out of 14 scored items (2 sequences compared, with 7 features analysed in each comparison) 3 T MRI tended to be superior to 7 T on none of the 14 items (Table 3). When combining all seven analysed characteristics on T2 and FLAIR in each individual patient, 7 T scored better than 3 T (Table 4).

**Table 3:**
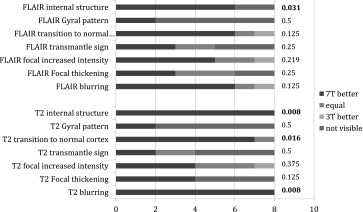
Conventional visual analysis of 3 vs 7 T images

*Blue* 7 T superior to 3 T, *red* 7 T and 3 T equal, *green* 3 T superior to 7 T, *purple* feature not visible, *Last column* *p*-values Sign Test, significant values in **fat**

**Table 4:** Comparison between 3 and 7 T MRI’s combining all characteristic on T2 and FLAIR

Patient	2	3	4	6	7	8	10	11	7 T better:3 T better
Blurring T2	1	1	1	1	1	1	1	1	8/8:0/8
Focal thickening T2	Na	0	1	1	Na	1	1	0	4/6:0/6 (na:2)
Focal increased intensity T2	1	0	1	1	1	0	0	−1	4/8:1/8
Transmantle sign T2	Na	0	Na	Na	Na	0	1	1	2/4:0/4 (na:4)
Transition to normal cortex T2	1	1	1	1	1	1	1	0	7/8:0/8
Gyral pattern T2	Na	Na	1	1	Na	Na	Na	Na	2/2 (na:6)
Internal structure T2	1	1	1	1	1	1	1	1	8/8:0/8
Blurring FLAIR	−1	0	1	1	1	1	1	1	6/8:1/8
Focal thickening FLAIR	Na	0	1	1	Na	1	0	0	3/6:0/6 (na:2)
Focal increased intensity FLAIR	−1	0	1	1	1	1	1	0	5/8:1/8
Transmantle sign FLAIR	Na	1	Na	Na	0	0	1	1	3/5:0/5 (na:3)
Transition to normal cortex FLAIR	−1	1	1	1	1	1	1	W 7 T, G 3 T (1)	6^a^/8:1^a^/8
Gyral pattern FLAIR	Na	Na	1	1	Na	Na	Na	Na	2/2:0/2 (na:6)
Internal structure FLAIR	Na	Na	1	1	1	1	1	1	6/6:0/6 (na:2)
Total 7 T better than 3 T	4/7	5/11	12/12	12/12	8/9	9/12	10/12	6/11^a^	
Total 3 T better than 7 T	3/7	0/11	0/12	0/12	0/9	0/12	0/12	1/11^a^	
Total not applicable	7	3	2	2	5	2	2	2^a^	

*−1* 3 T better than 7 T, *0* equally visible on 3 T and 7 T, *1* 7 T better than 3 T, *Na* Not applicable as not visible on either 7 T or 3 T, *W* white matter, *G* gray matter

^a^Excluding patient 11 transition to normal cortex on FLAIR

**Fig. 6:**
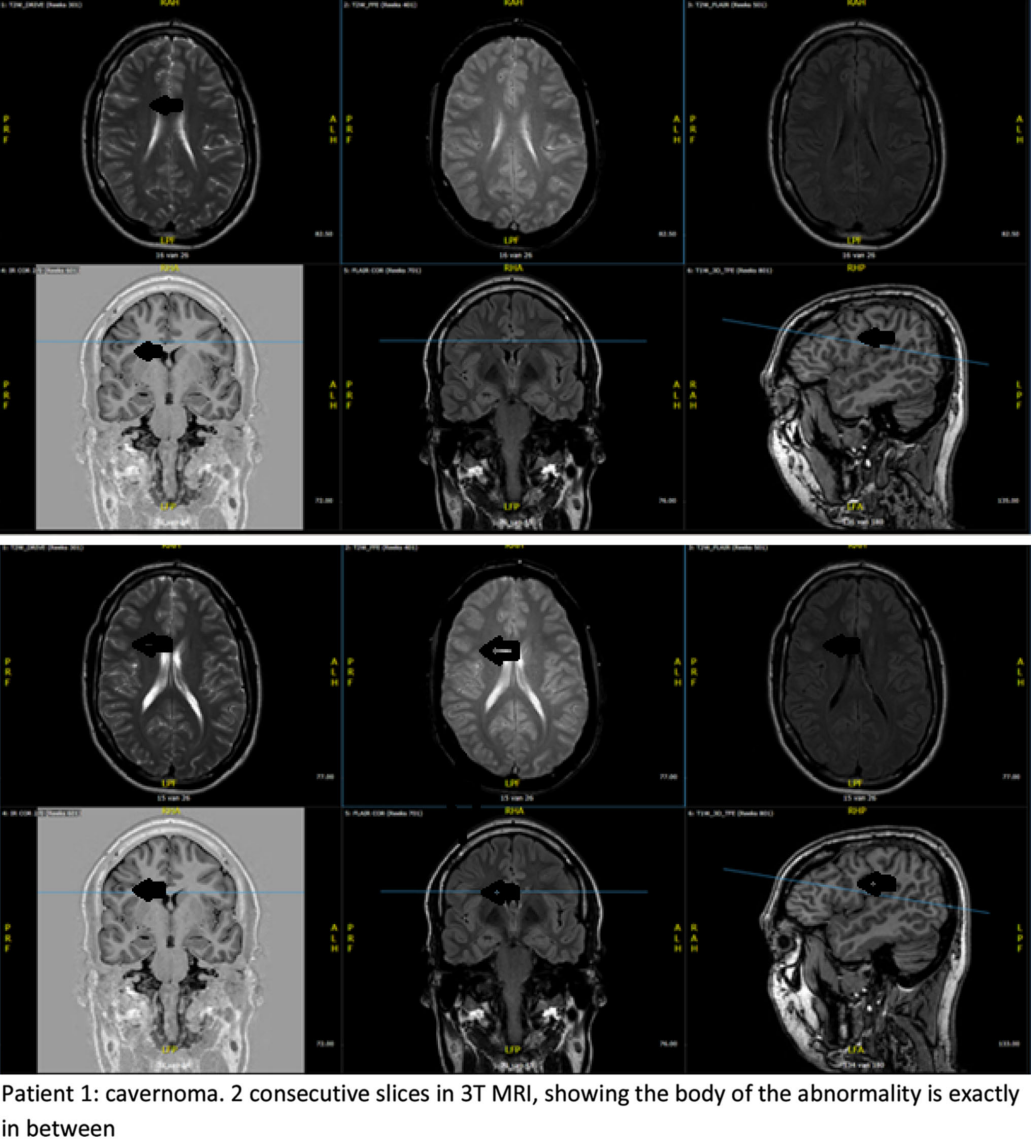

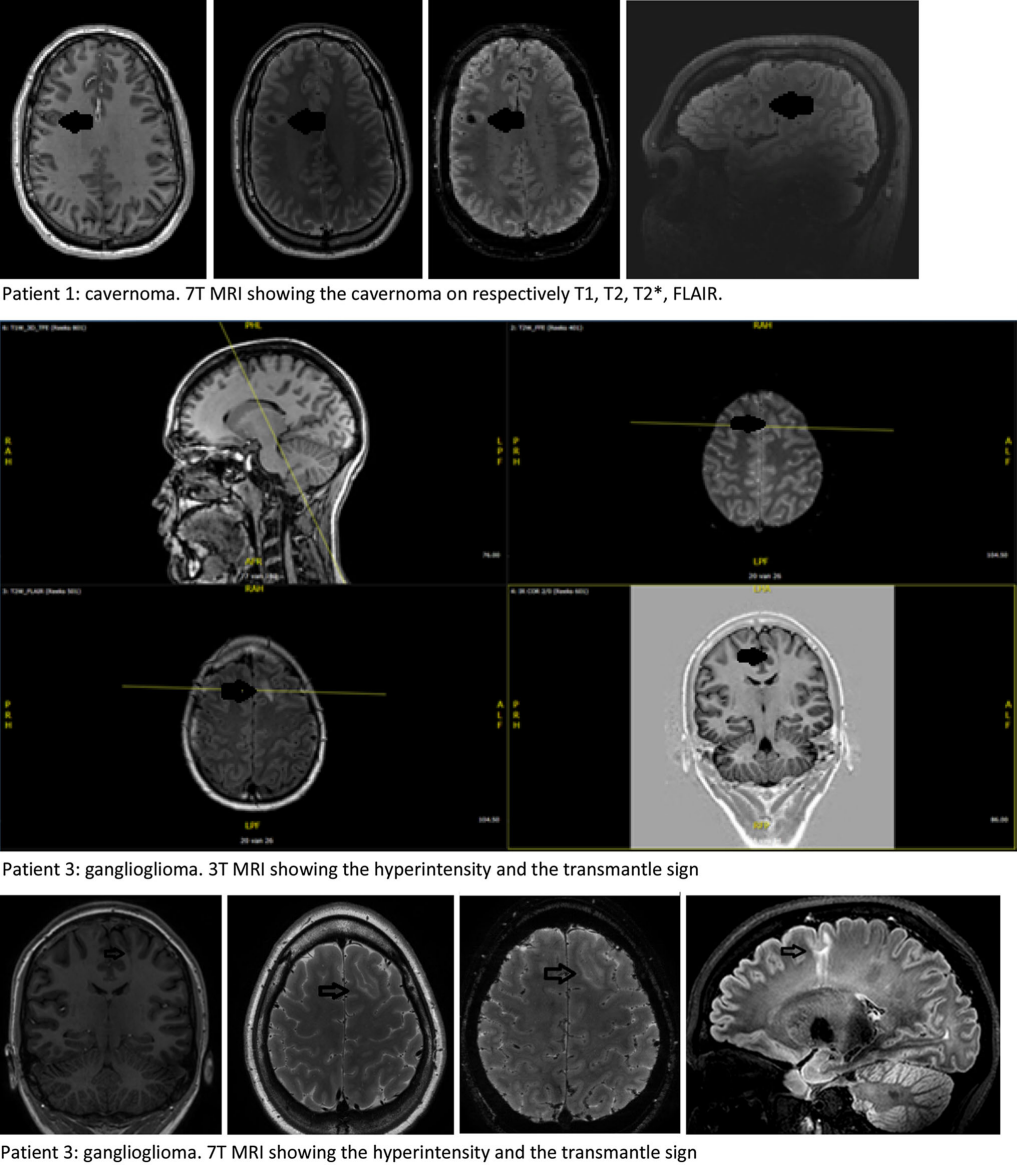
3 and 7 T MRI images of the two patients with changed diagnosis. Each sequence contrasted and angulated individually

Five of the six included patients that were also evaluated for epilepsy surgery were operated. In patient 3 histopathology showed a ganglioglioma WHO grade 1, patient 8 had FCD type IIIb (frontal infantile desmoplastic ganglioglioma with bordering FCD operated plus parietal FCD that we analysed in this study), patient 9 showed a FCD type IIa and patients 10 and 11 showed a FCD type IIb. The location of the lesion was congruent between MRI and surgical specimen. The 3 and 7 T MRI images of the two patients in whom the diagnosis changed from FCD to, respectively cavernoma and ganglioglioma are shown in Fig. 6.

One patient complained of profound nausea at entering the MRI. Slowing down the table movement reduced the symptoms. During the imaging this complaint was not present.

## Discussion

The main finding of this study is that all observers agreed that on conventional visual analysis the lesions were easily detected and better detailed with the applied 7 T MRI protocol than on lower field strength. None of the known lesions were missed on the 7 T images. Using a semi-quantitative scale, overall our 7 T MRI protocol tended to be superior to the previously applied 3 T protocol. Statistical significance was reached for 4 out of 14 scored items. In two patients final diagnosis changed from FCD to, respectively cavernoma and gangioglioma.

As far as we know this is the first publication describing 7 T MRI in a group of patients with suspicion of FCD using a standard clinical protocol.

In one patient, due to the 7 T images diagnosis changed from FCD to cavernoma. There are several explanations possible for this change. First, due to slice thickness in the 3 T images the small hemosiderin deposit could have been located exactly in between two slices, thus escaping detection. Due to the thinner slice thickness of the 7 T images, the hemosiderin is more obvious. The images as depicted in Fig. 6 seem to substantiate this hypothesis. Second, the artefact effect of hemosiderin is more pronounced on 7 T MRI than on 3 T MRI, thereby highlighting the cavernoma more evidently in 7 T MRI. The sequence most sensitive for hemosiderin is SWI (susceptibility weighted imaging). However, in epilepsy the presence of small hemosiderin deposits is of minor relevance and therefore in the initial phase of our study this sequence was not part of our standard epilepsy protocol. This will have probably lowered the sensitivity for the detection of small haemorrhages of the 3 T MRI more than the sensitivity of 7 T MRI. Especially compared to 7 T MRI in which a T2* weighted sequence was part of the protocol. Third, there is a time delay of several months between these 3 and 7 T MRI’s. Although there were no additional clinical symptoms, it is possible that in between these time points the amount of blood surrounding the cavernoma increased. As the patient is no longer under our care we regrettably do not have access to a 3 T MRI made after the 7 T MRI.

In one of the five operated cases histopathology showed that the abnormality was a ganglioglioma instead of a FCD type II as the radiological diagnosis stated. Re-challenging of three pathologists and two radiologists with information on the opinion of the other specialist did not change their conclusions. Taking the diagnosis of the pathologists as golden standard, this proves that MRI can help giving an indication of the diagnosis. But visual inspection is not (yet) able to provide the definite diagnosis with 100 % certainty. Noteworthy is the fact that in this patient (patient 3) all evaluated MRI-characteristics of FCD were present.

The frequency of the imaging features of FCDs as seen in our study is higher than reported for FCD in the literature for 1.5 and 3 T studies. For example cortical thickening was seen in 50 % [29] to 76 % [30] whereas in our series it is 89 %. Blurring ranged from 36 % [31] to 87 % [32], whereas in our series it is 100 %. For transmantle sign this ranged from 19 % [33] to 81 % [31], and in our series it is 67 %. Most publications are based on histopathologically proven diagnoses of FCD, including patients without MRI abnormalities, whereas for our study the suspicion of a FCD on lower field strength MRI was an inclusion criterion. This explains the relative high frequency of the imaging features in our series. Quality of the 7 T MRI images itself plays a role as well: signal-to-noise ratio scales approximately linear with magnetic field strength [34, 35]. Furthermore, it has also been observed that 7 T MRI provides an increased contrast-to-noise ratio in FLAIR as compared to 1.5 and 3 T [13, 36]. The smaller voxels that can be achieved with 7 T MRI within a clinically applicable protocol will also provide better spatial resolution, leading to the detection of thin abnormalities, such as blurring or the transmantle sign.

Other limitations of the current studies include the fact that, besides the magnetic field strength, the scanners differed with respect to other hardware such as the number of receive channels of the head-coil and that choices of sequence parameters were based on local expertise without an effort to homogenize these between the field strengths. Comparisons were made between acquisitions made on the scanners available to us, which led to inherent differences in receive and transmit coil properties, other hardware components as well as software. Using, for example, a 32-channel head coil for the 3 T might have improved image quality on that field strength. However, based on our experience with both field strengths we think that magnetic field strength is the main contributor to the observed improved image quality.

Applying visual analysis, in our series 7 T MRI FLAIR was the sequence on which the lesions were most prominent. The flag-like three-layer appearance is easiest appreciated on T2. The middle hypo-intense line is accentuated by the bordering hyper-intense parts of the lesions. However, even though much less pronounced, looking at the homologue contralateral area this line often is bilaterally noticeable. The appearance of this line is not equally distributed in all different regions, which is in line with the findings of Zwanenburg et al. [37] who described similar regional differences (but no asymmetries) in the normal brain in 7 T MRI. The line is located at the grey–white matter junction and is present in almost all regions, and therefore it does not represent the striae of Gennari [38]. Based on our small series, this line seems to be more prominent with rising age, even more in the FCD-like region than in the other regions of the brain. We postulate that this line represents iron deposits which would explain an age-dependency [39]. Alternatively, this line could represent a low signal coming from the deepest cortical layer, seen on thin 7 T slices but masked on thicker 3 T slices. In 7 T MRI this accentuated three-layer appearance on the T2 weighted images has the potential to be used as an imaging marker of FCD. The internal structure and extend of the lesions were best visible on T2 and especially T2* sequences. This is in line with expectations, as the T2* sequence provides high spatial resolution and sensitivity to the magnetic susceptibility properties of tissues, thus improving evaluation of the different components within the cortex. When in more cases histopathology will become available, this might help in differentiating between different pathological substrates, like the different types of FCD.

Although due to the nature of this study we did not co-register all the sequences of 3 and 7 T study, visual inspection and interpretation support the notion that on 7 T the lesion seemed often to extend beyond what is seen on 3 T.

Because of the better delineation of lesions on 7 T, if intracranial EEG recording is needed we would advise to use the T2 sequence to guide the implantation. Especially in case of multiple depth-electrodes (stereo-EEG) where presurgical delineation of the abnormality is even more dependant on electrode placing than when using grids.

In epilepsy, abnormalities observed on MRI do not always reflect the epileptogenic focus. This is illustrated by the observation of Salmenpera et al. [40] that 9 % of 3 T MRI-positive findings are not related to the epileptogenic lesion. Therefore, every positive MRI result should be interpreted with caution and in combination with the electro clinical findings. However, clinical assessment based on the intra-individual stereotyped semiology makes it likely that all our patients had a single epileptogenic focus, correlating well with the observed location of the lesion. Interpretation of MRI should only be done including all clinical information available. This holds true for all other modalities such as PET, MEG and intracranial EEG as well. Probably, multimodality fusion will increase insight in analysing difficult surgery cases. The limitation of our study is that we included only patients with a diagnosis of FCD based on 3 or 1.5 T MRI and only three patients had a histologically proven diagnosis. Further studies will evaluate the additional value of 7 T MRI in presurgical analysis in patients without abnormalities on 3 T MRI. There is one study on the detectability of FCD’s on 7 T MRI in 21 patients without lesions on 3 or 1.5 T MRI, showing a 29 % diagnostic gain [41]. Agreement in imaging interpretation was reached through consensus-based discussions based on visual identification of structural abnormalities. Four out of the six patients with a thus detected lesion were operated, all showing a FCD on histopathology. These results are almost identical to our own results [42].

## Conclusion

7 T brain imaging in vivo is feasible in epilepsy patients and can be beneficial. Lesions are well recognizable and details are better visible than at lower field strengths. The presence of typical FCD-characteristics on MRI; however, does not always reflect the final histopathological diagnosis.

## Electronic supplementary material

Below is the link to the electronic supplementary material.

Supplementary material 1 (DOCX 4141 kb)

Supplementary material 2 (DOCX 4995 kb)

Supplementary material 3 (DOCX 951 kb)

Supplementary material 4 (DOCX 2749 kb)

## Abbreviations

EEG: Electro encephalography
FCD: Focal cortical dysplasia
FLAIR: Fluid attenuated inversion recovery
IR: Inversion recovery
MRI: Magnetic resonance imaging
PET: Positron emission tomography
SWI: Susceptibility weighted imaging
T: Tesla
TE: Echo time
TR: Relaxation time
