# Supplementary figures and images for: Detection superiority of 7 T MRI protocol in patients with epilepsy and suspected focal cortical dysplasia
Source: Acta Neurol Belg. 2016 Jul 8;116:259–69. doi: 10.1007/s13760-016-0662-x (PMC4989014; doi:10.1007/s13760-016-0662-x)

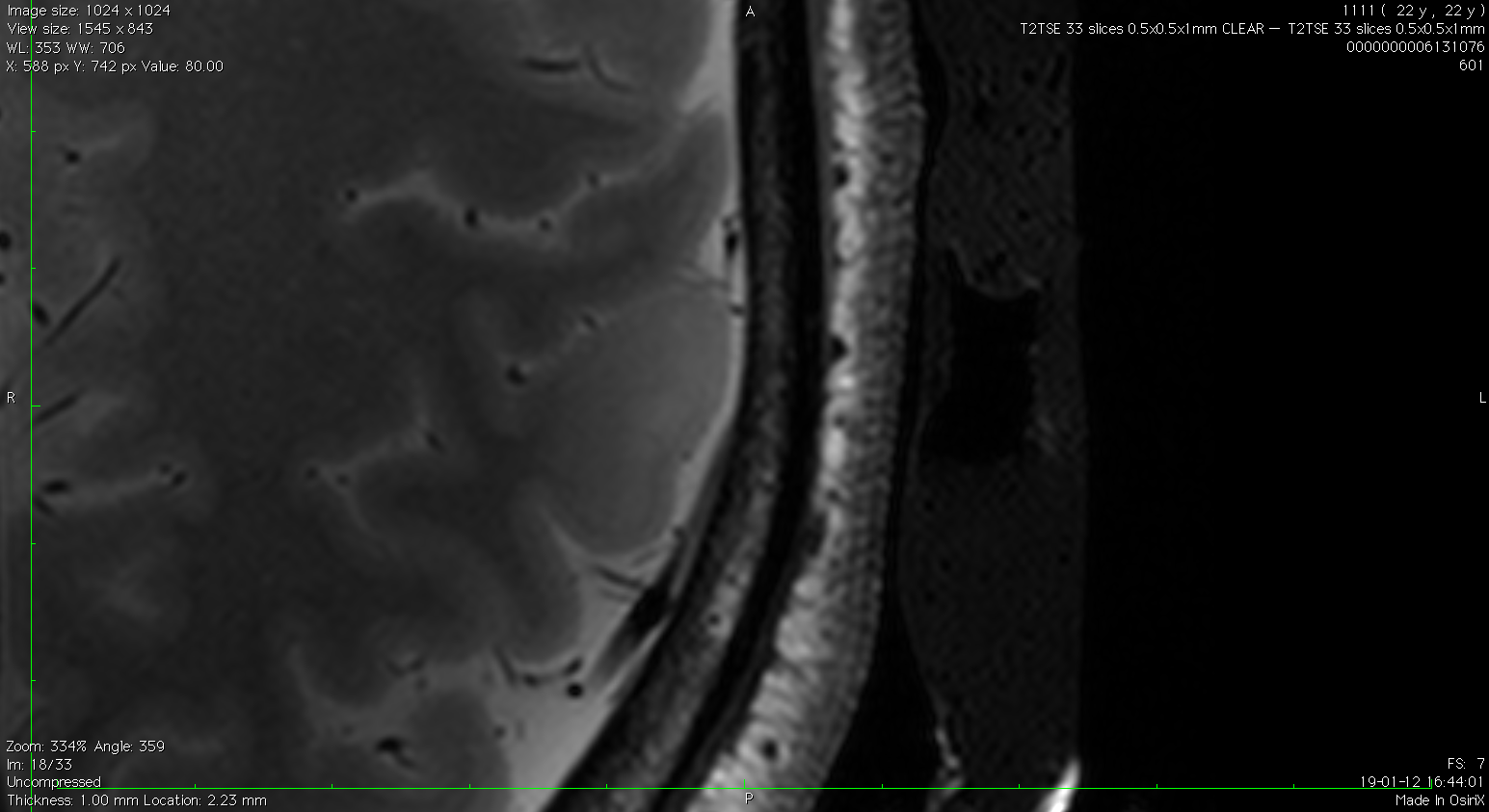

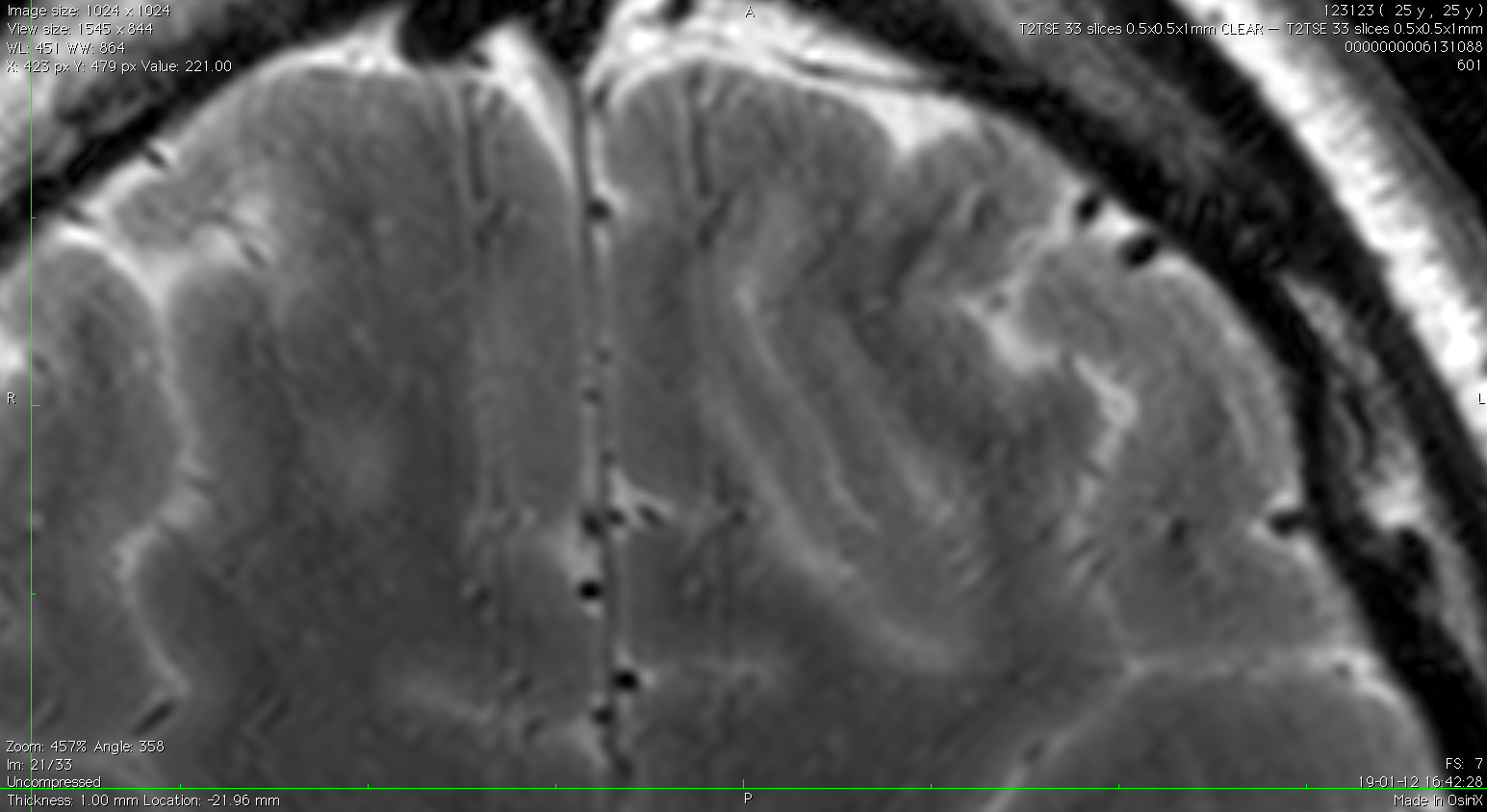


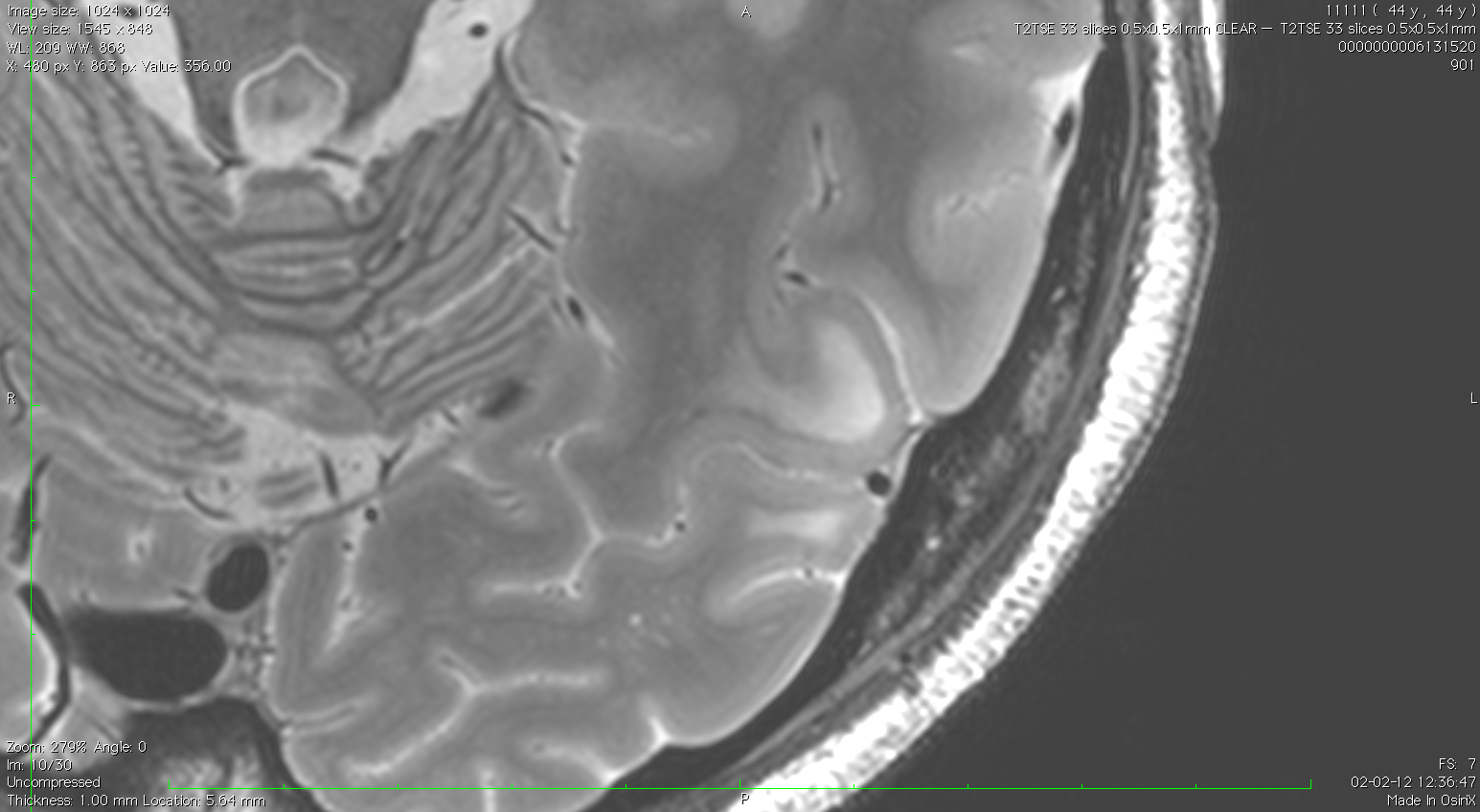

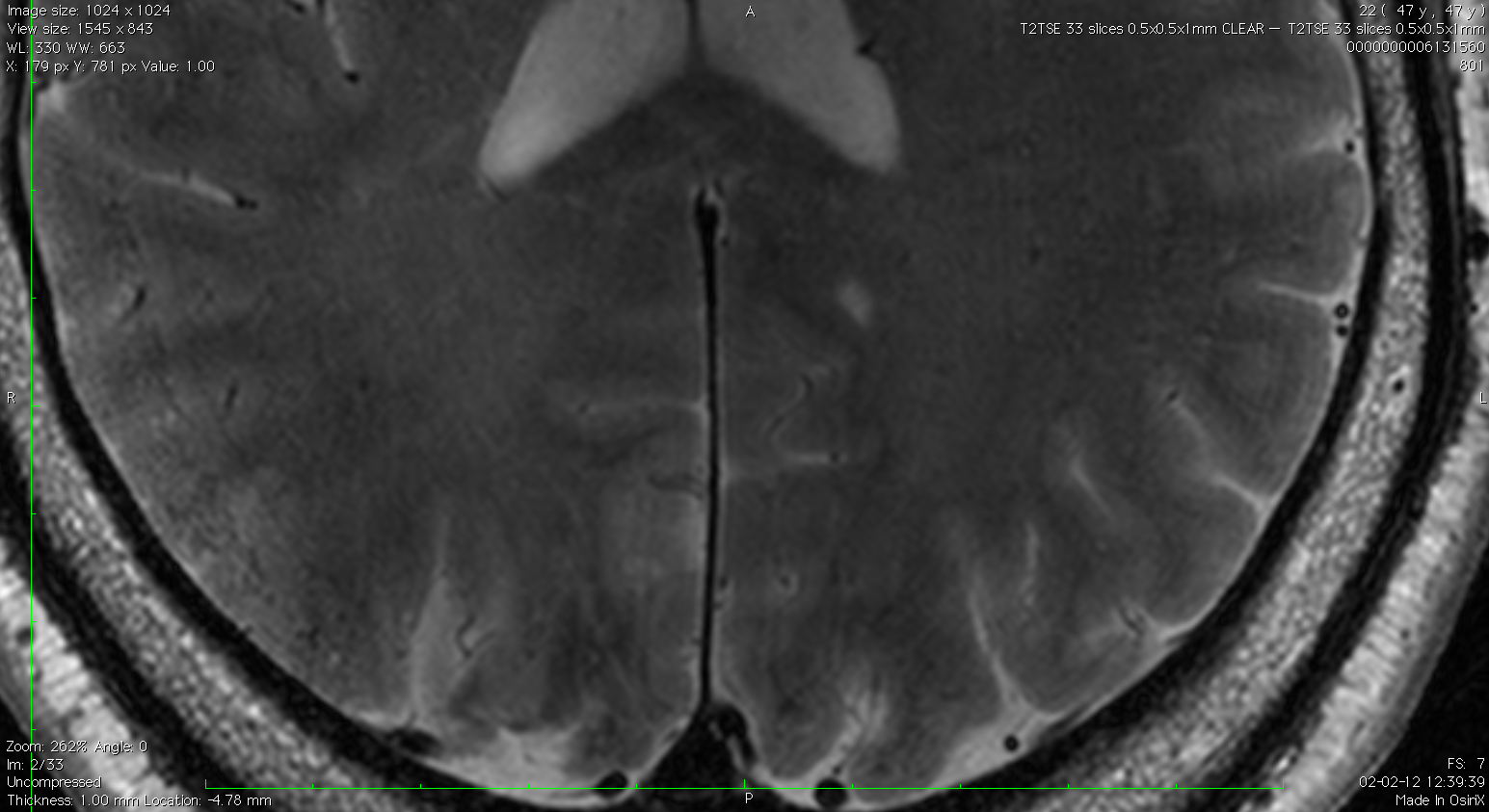


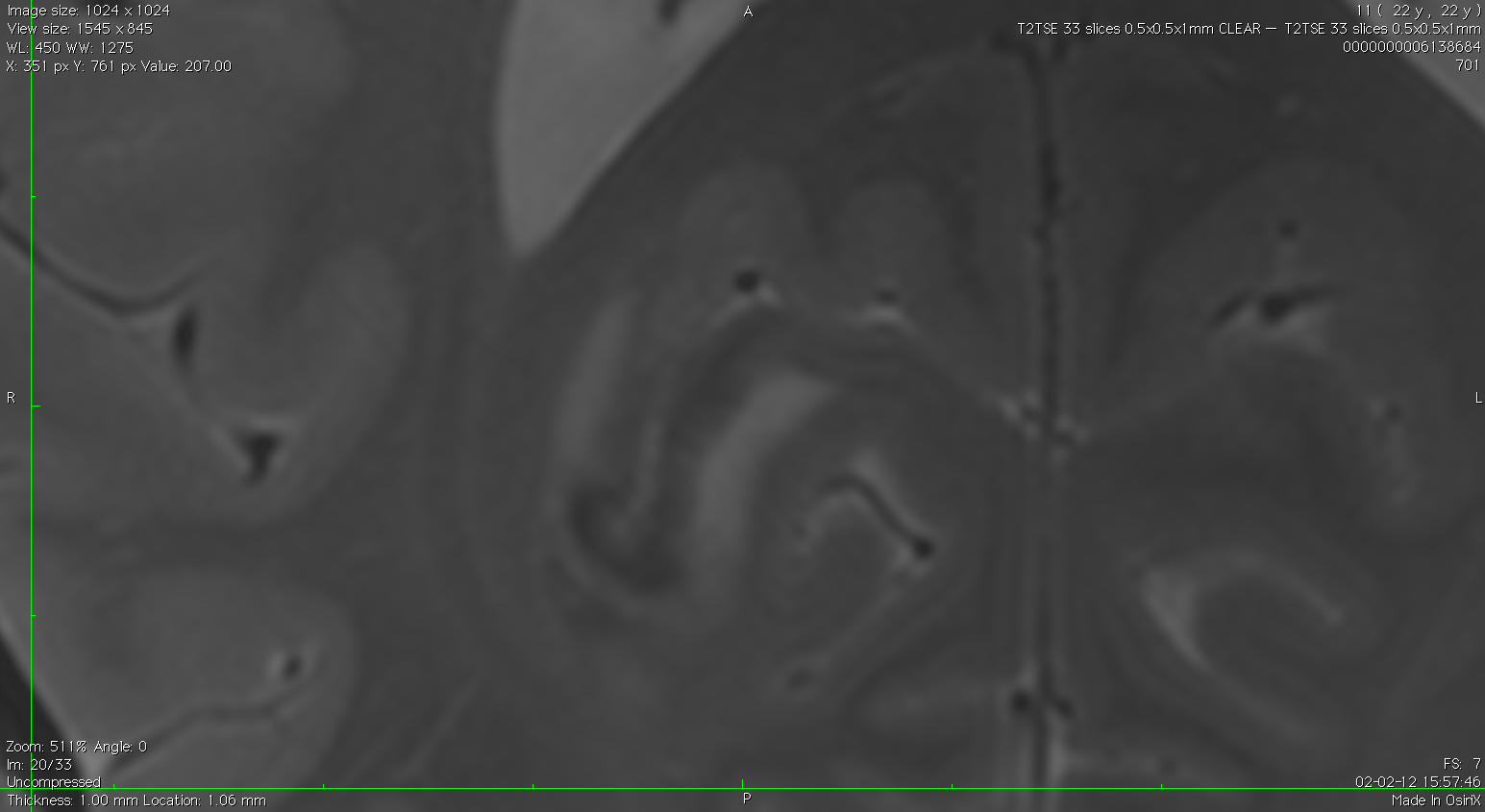

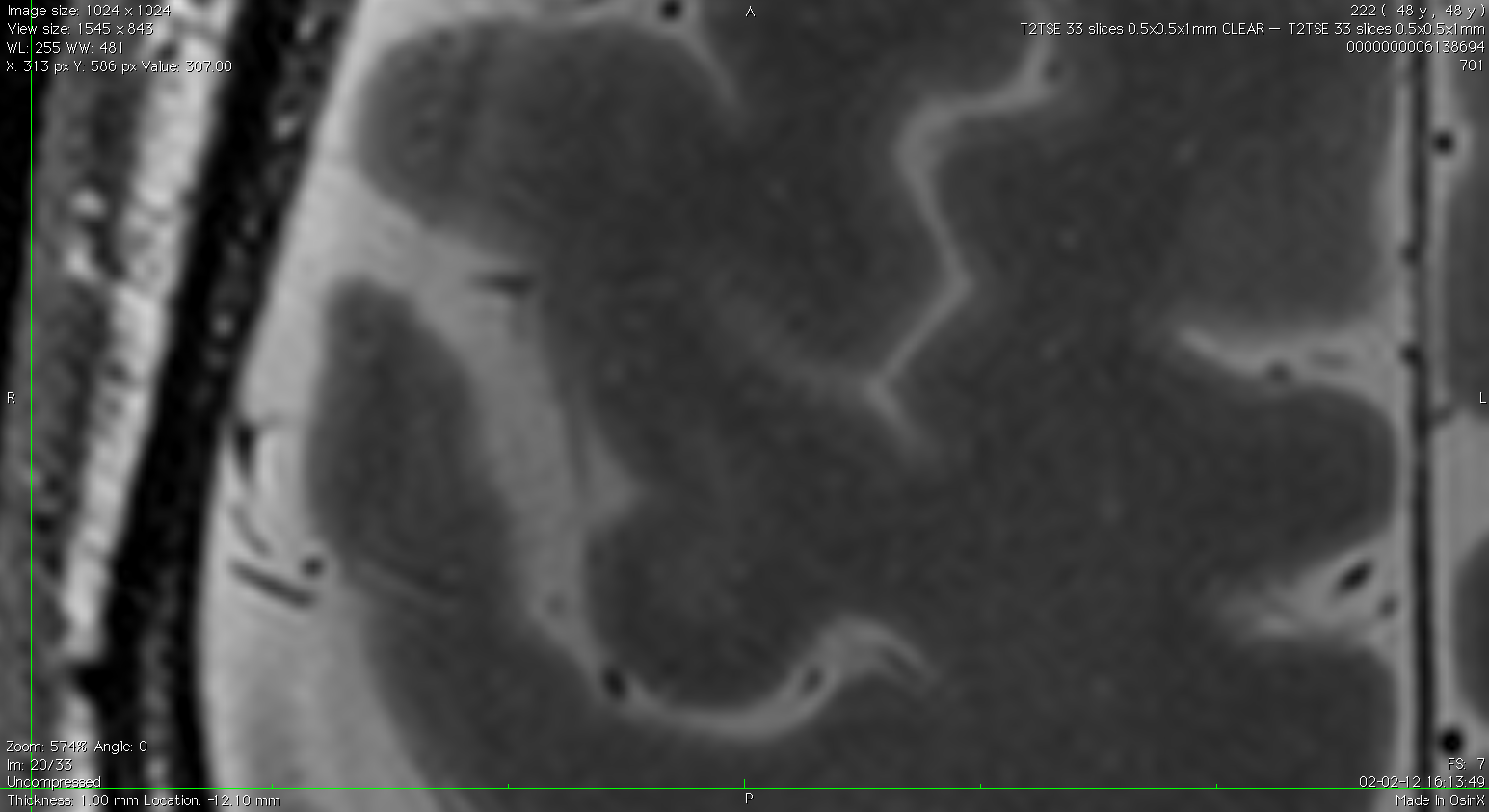


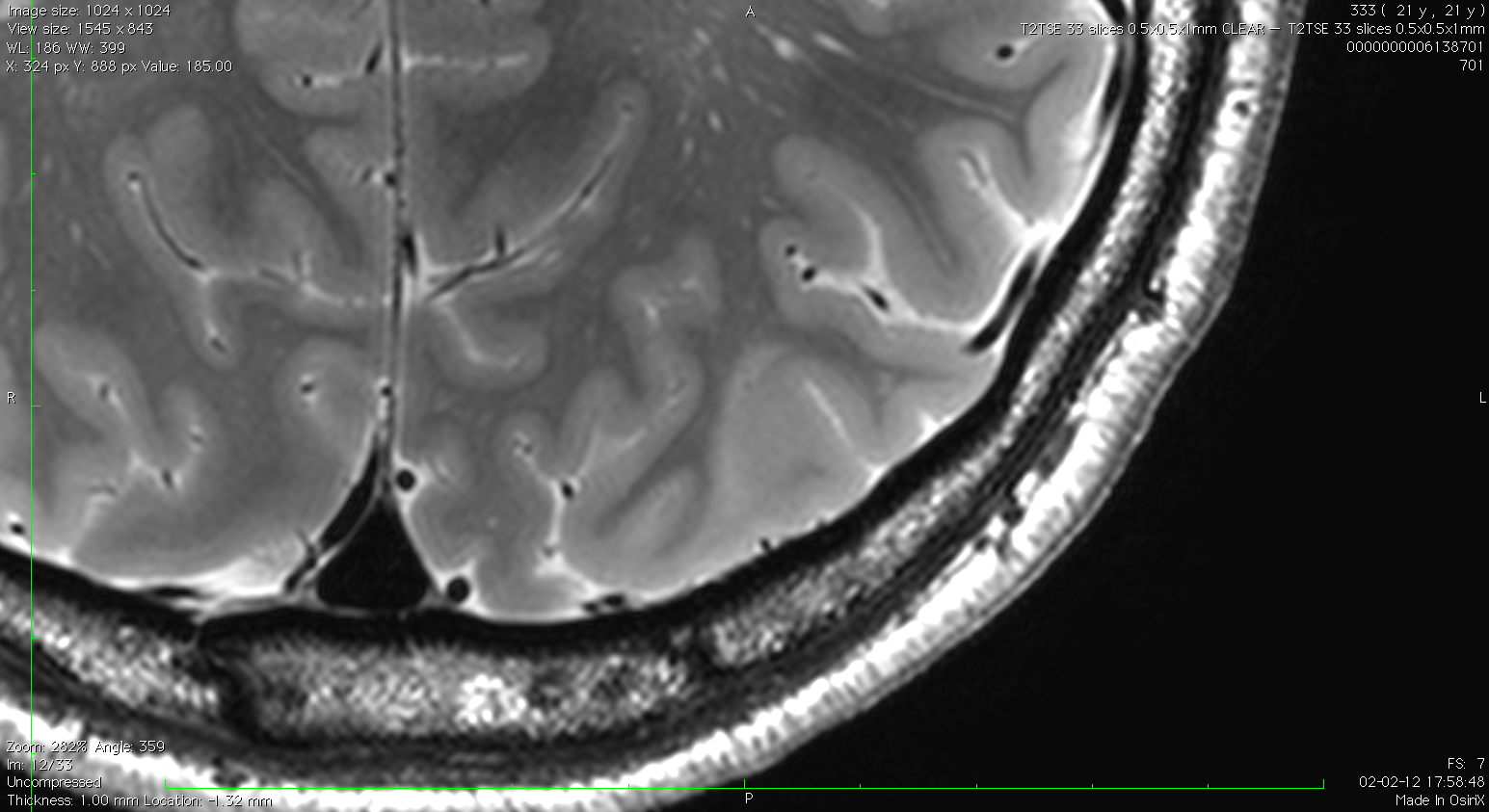

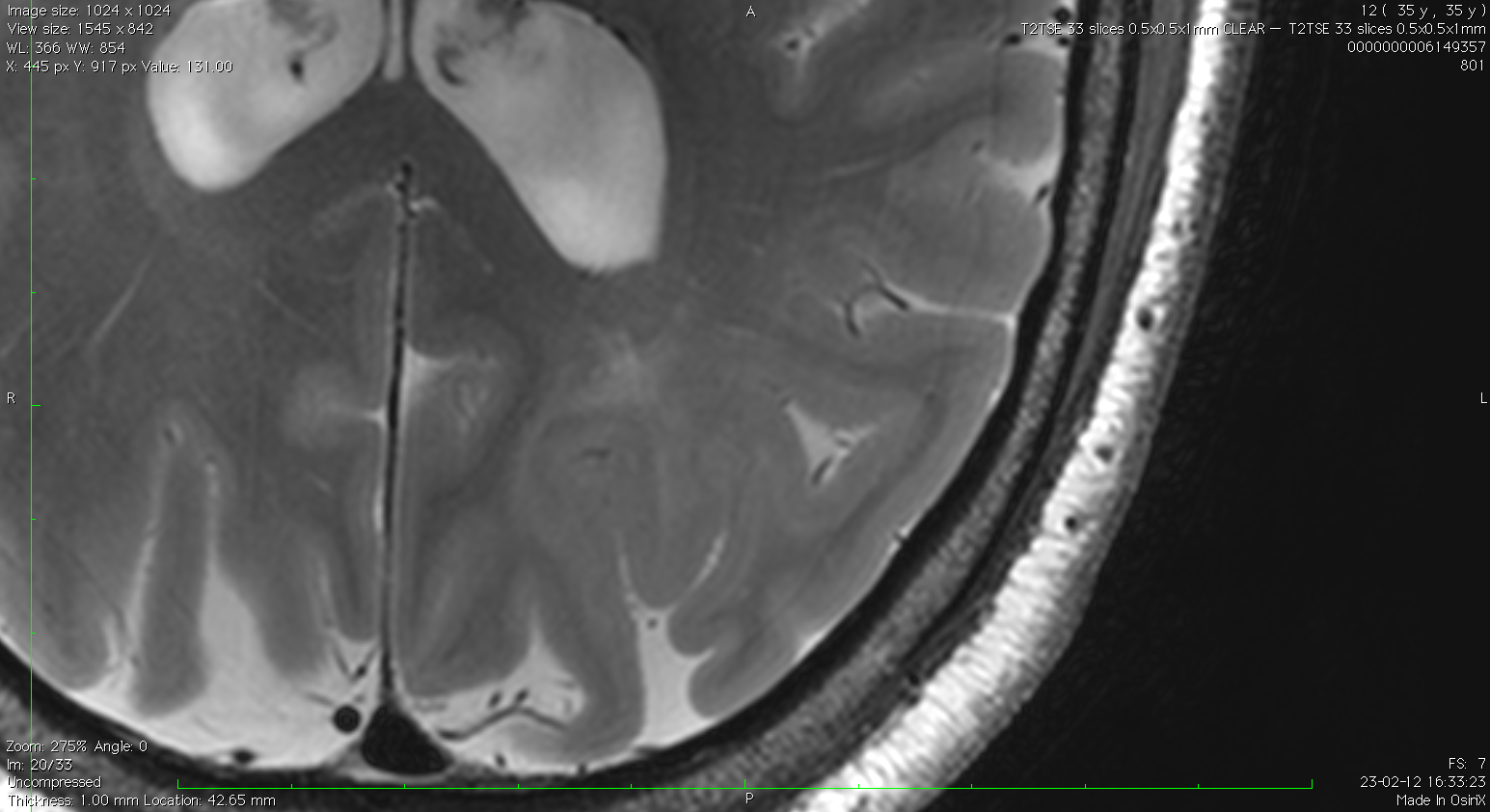


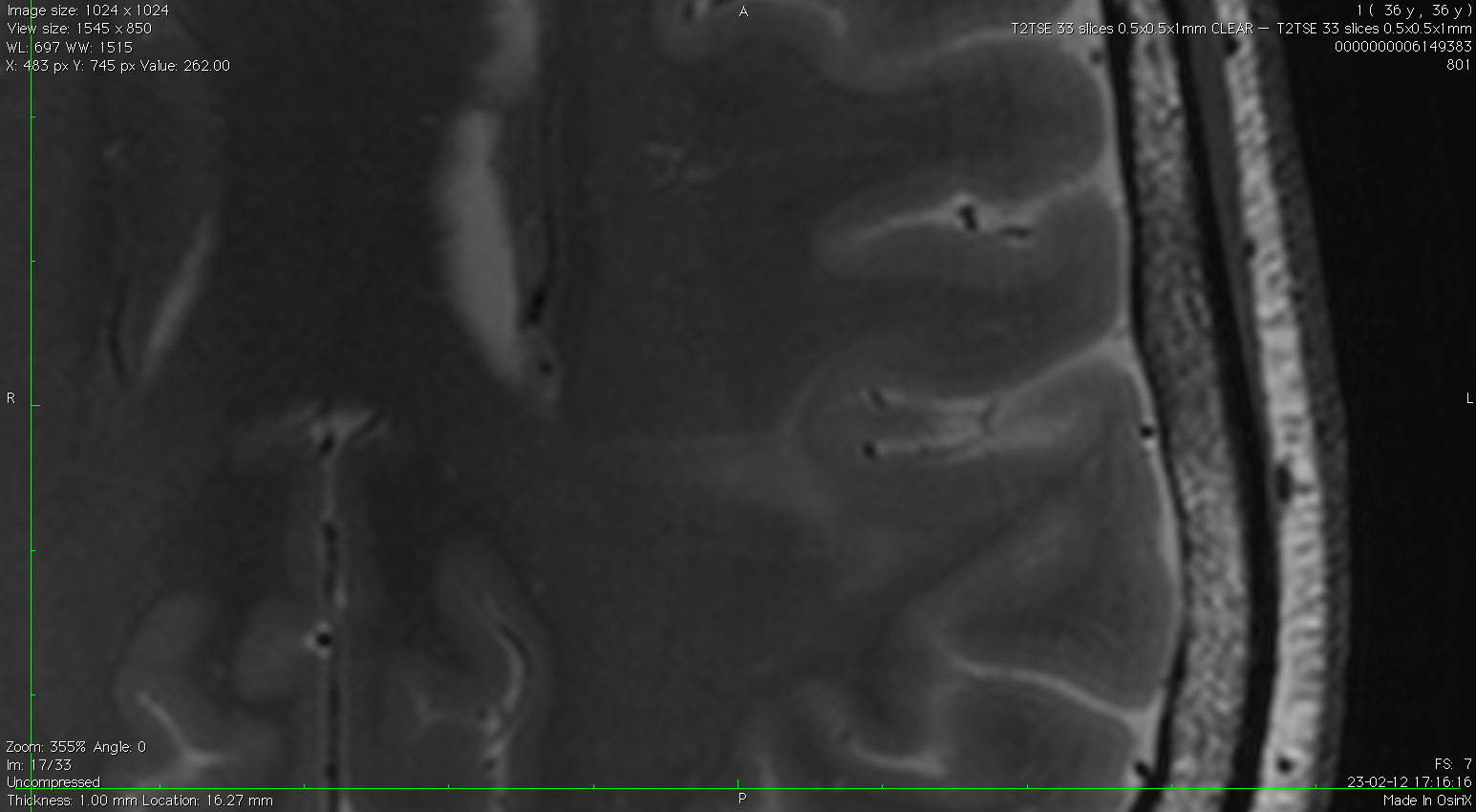

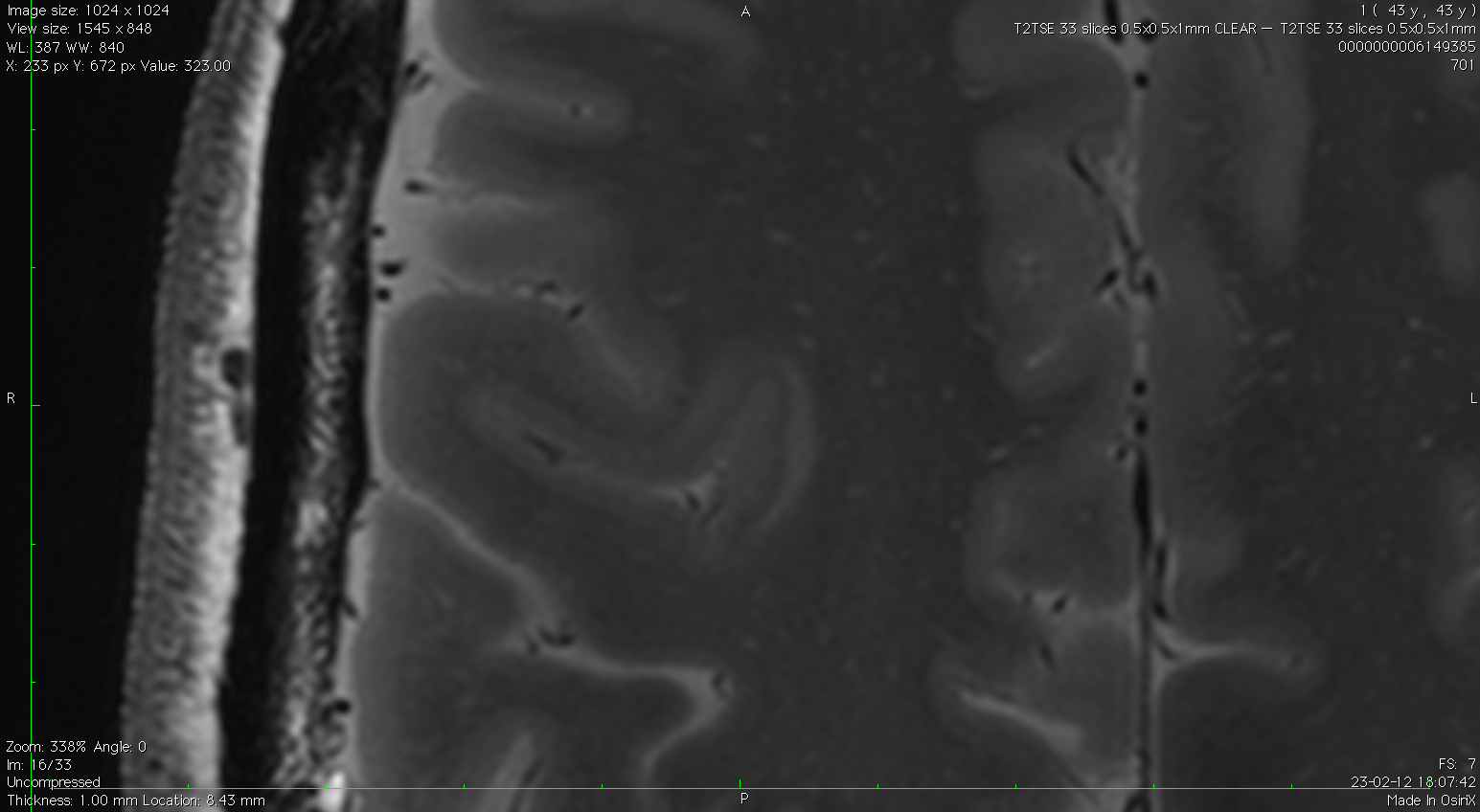


Supplement figure 2: Patient 2 to 11: Details of T2-weighted images of FCD on 7T

Supplement: Supplementary file 1 — Supplementary material 1 (DOCX 4141 kb) [file 13760_2016_662_MOESM1_ESM.docx]

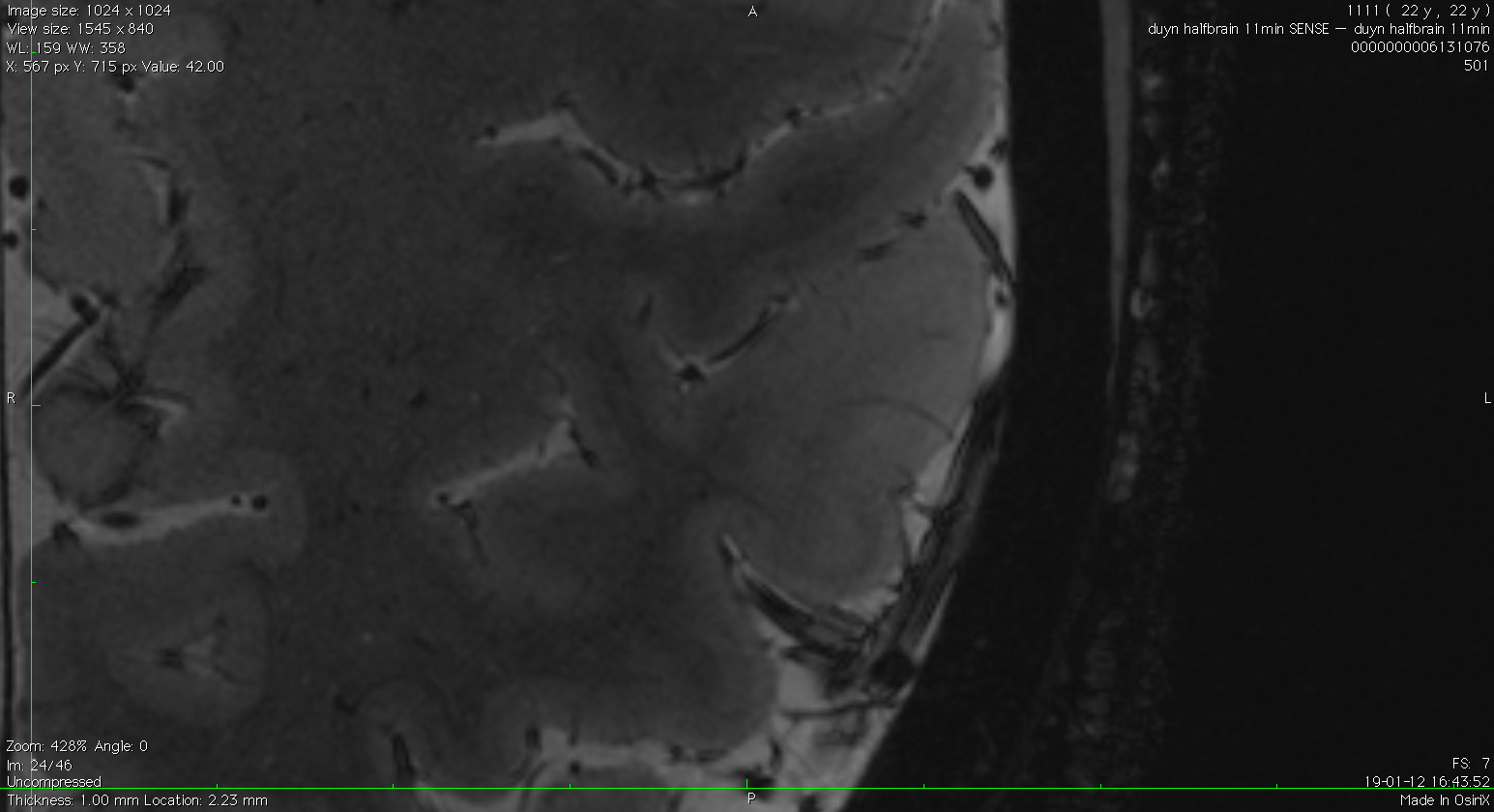

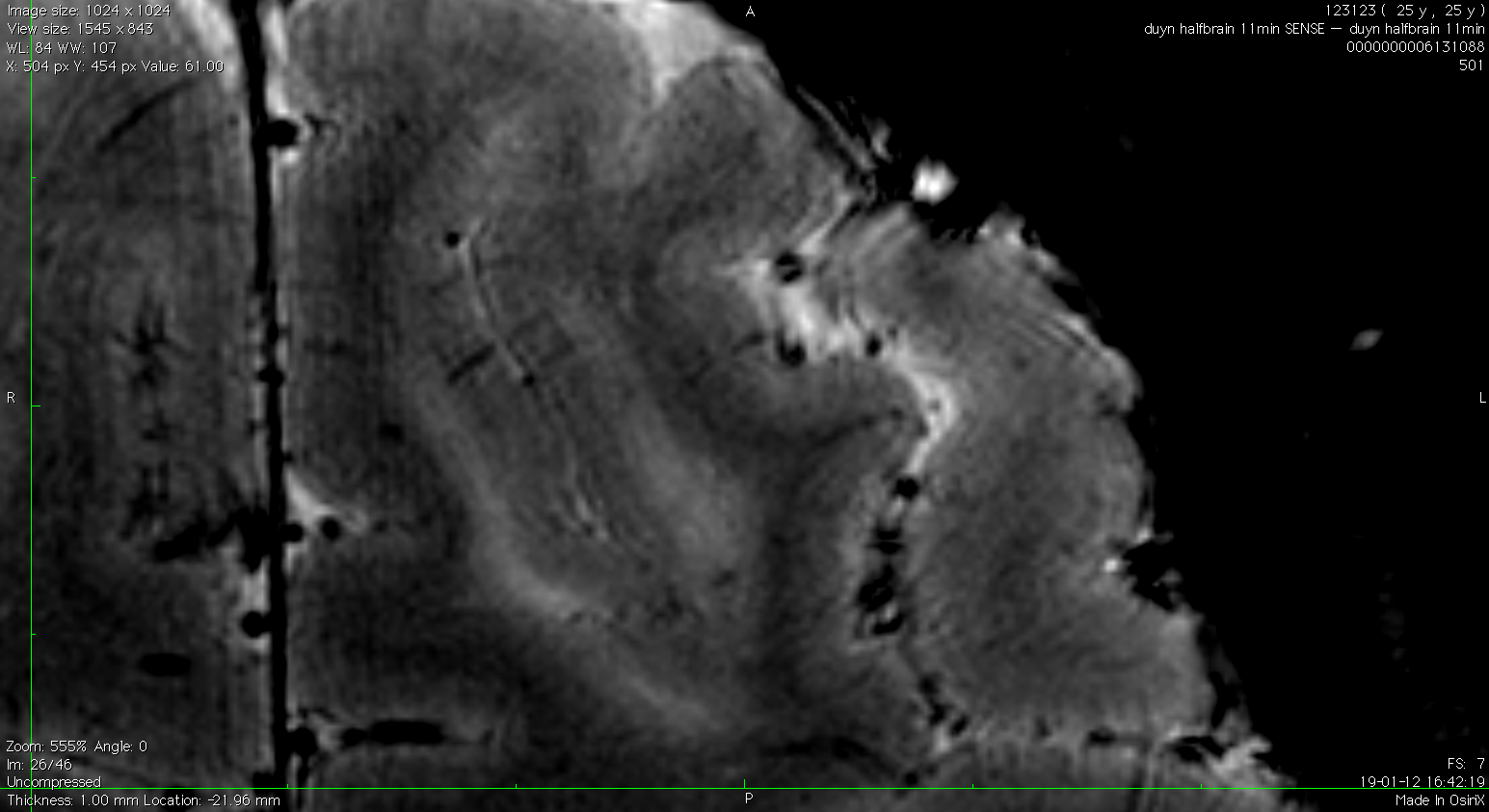


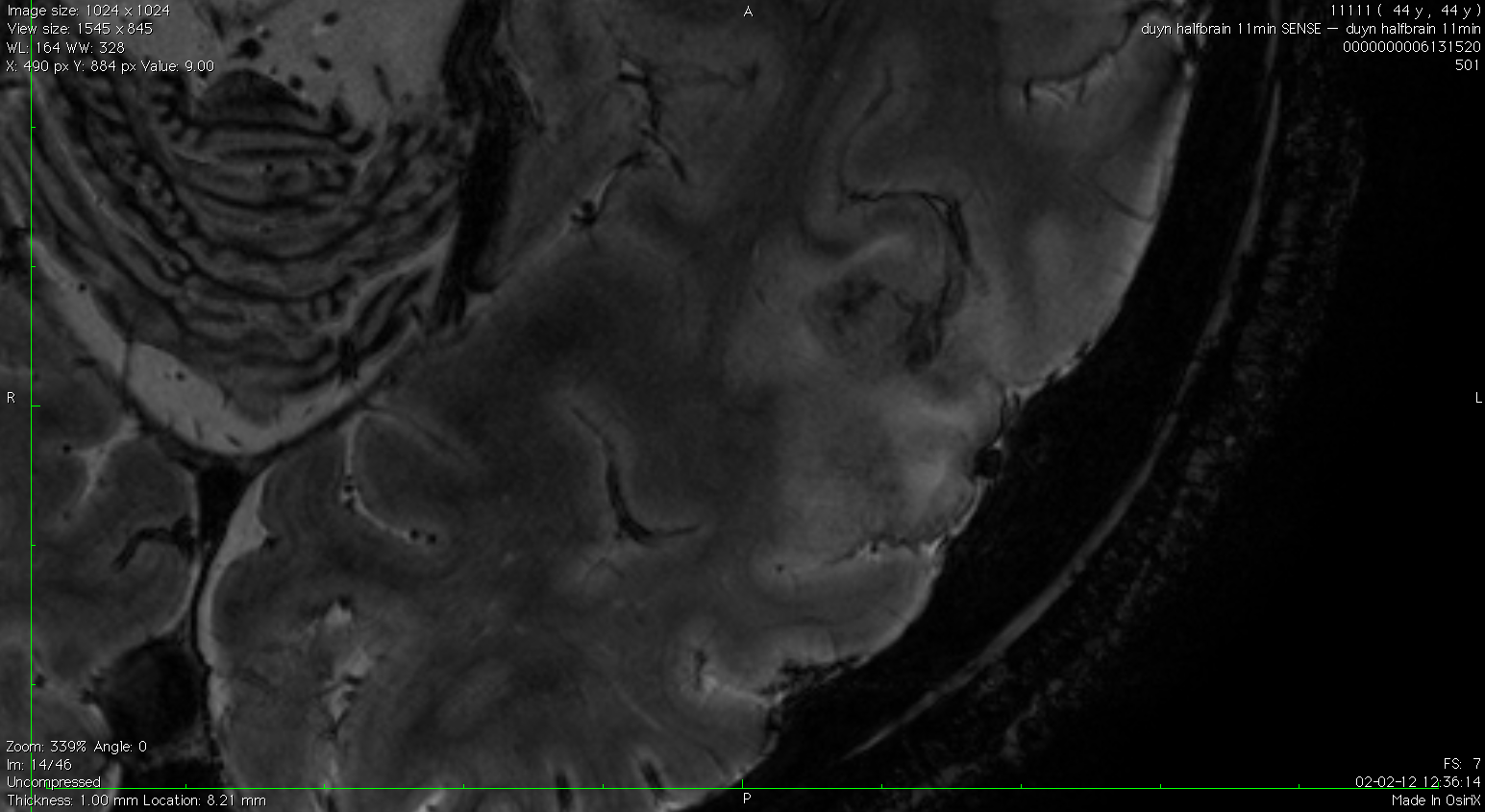

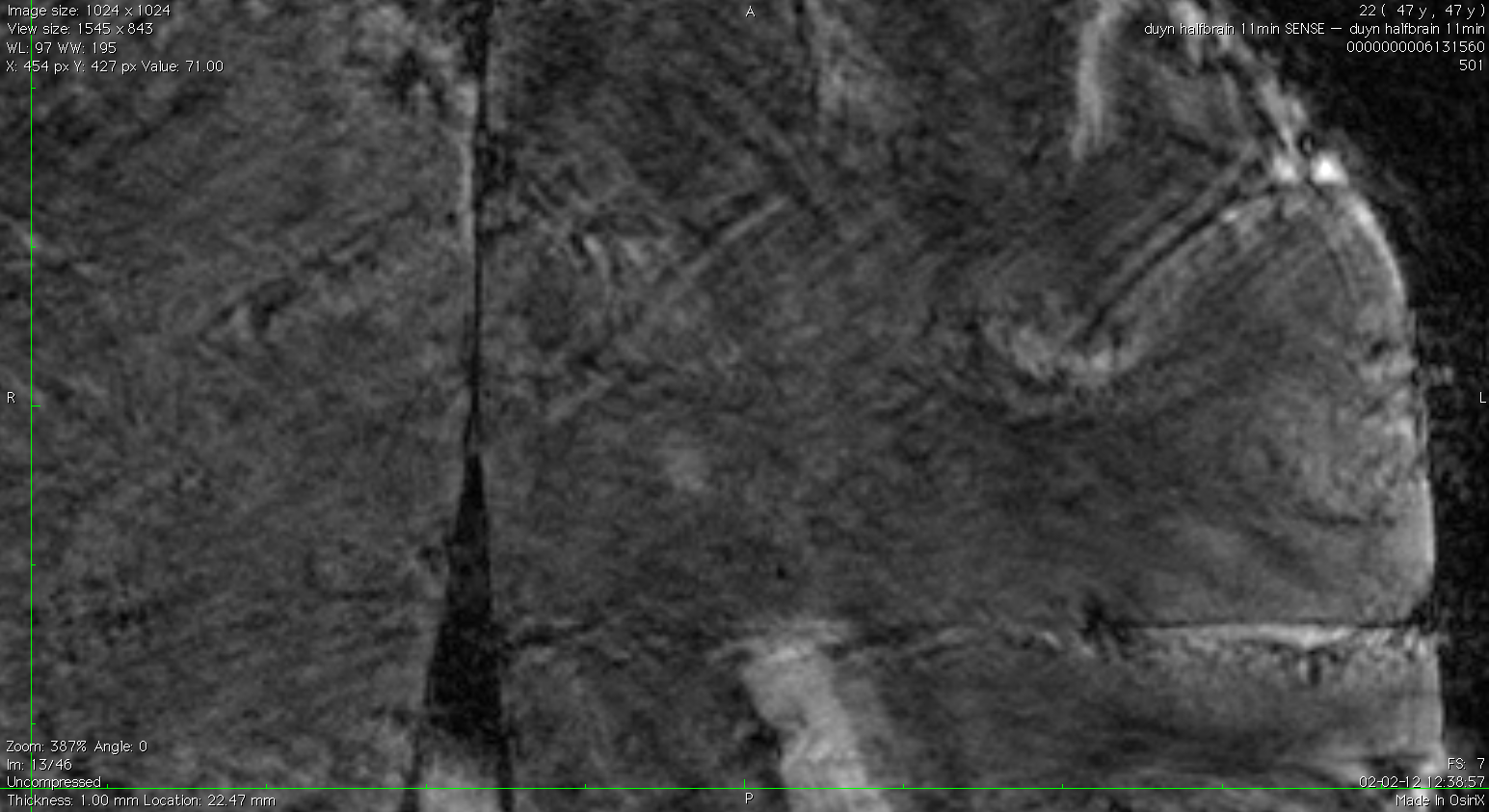


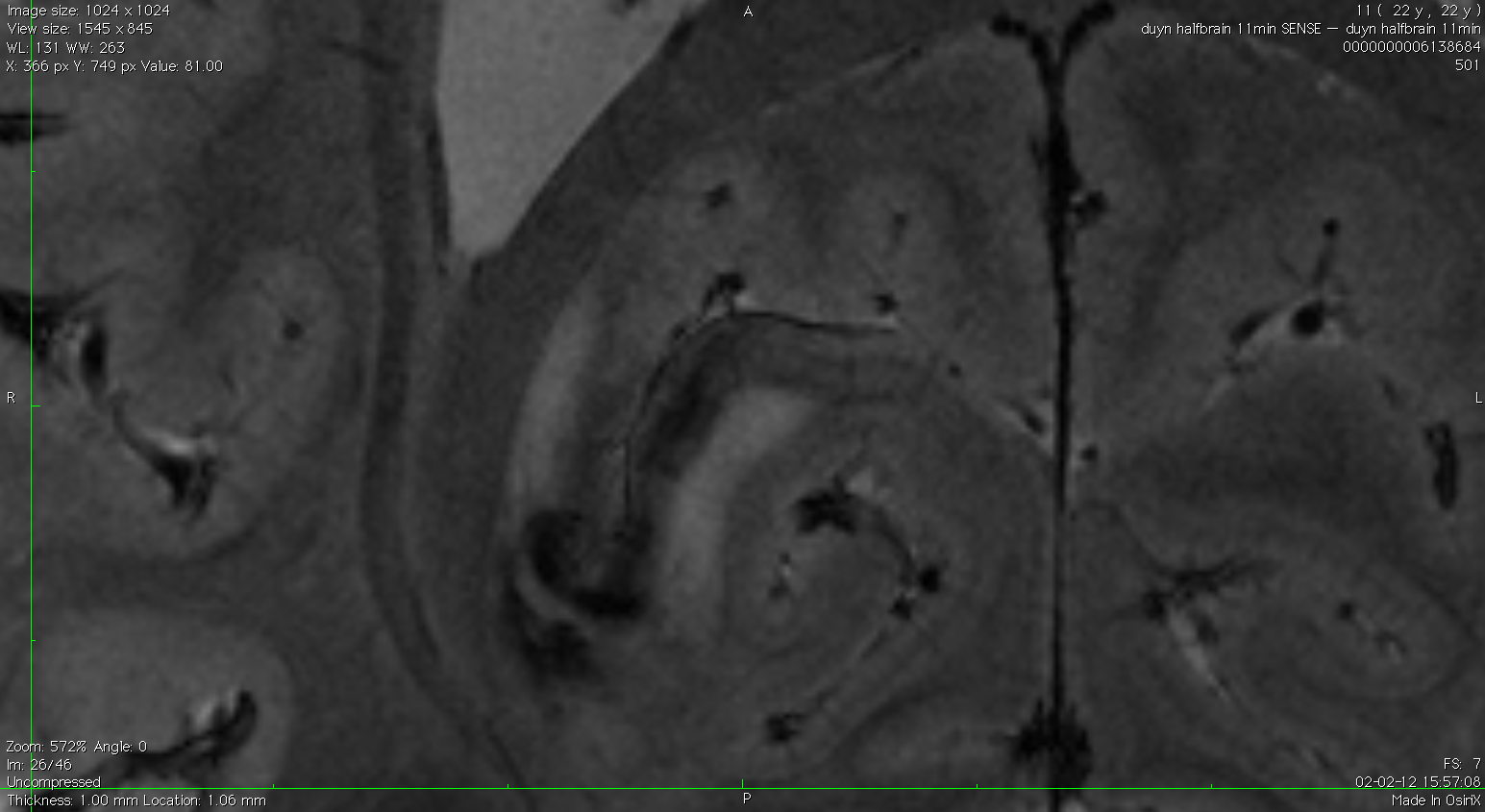

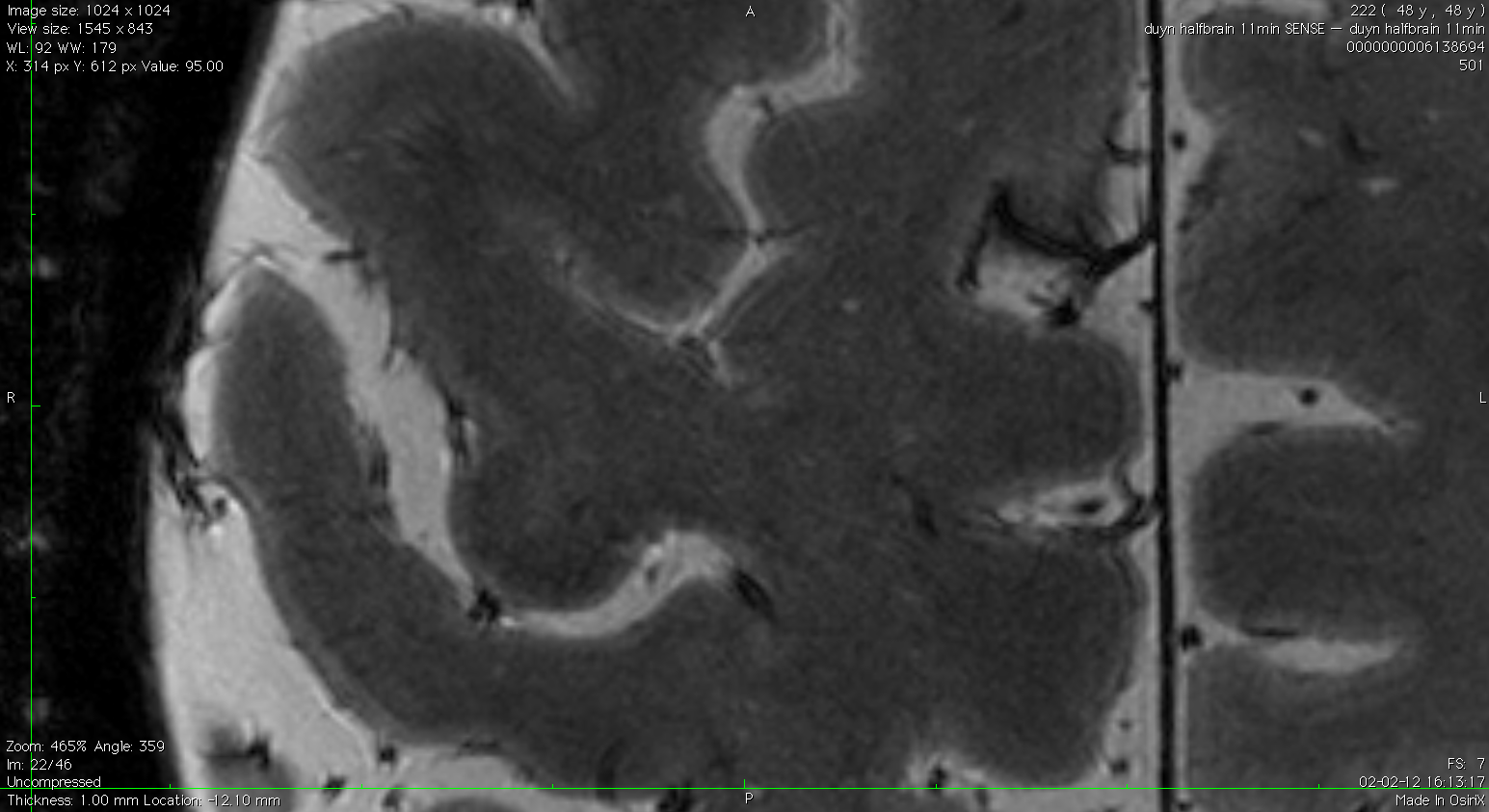


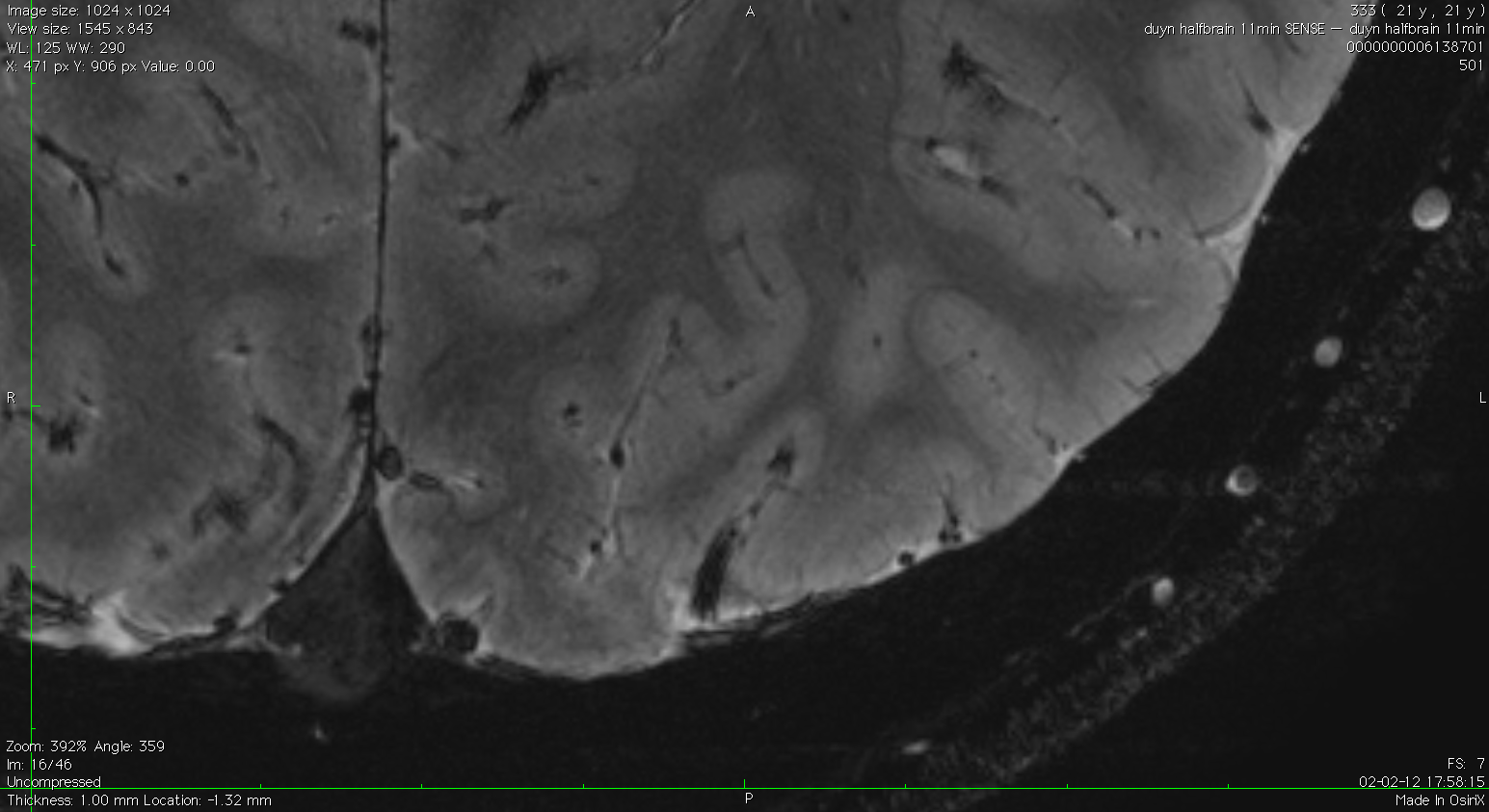

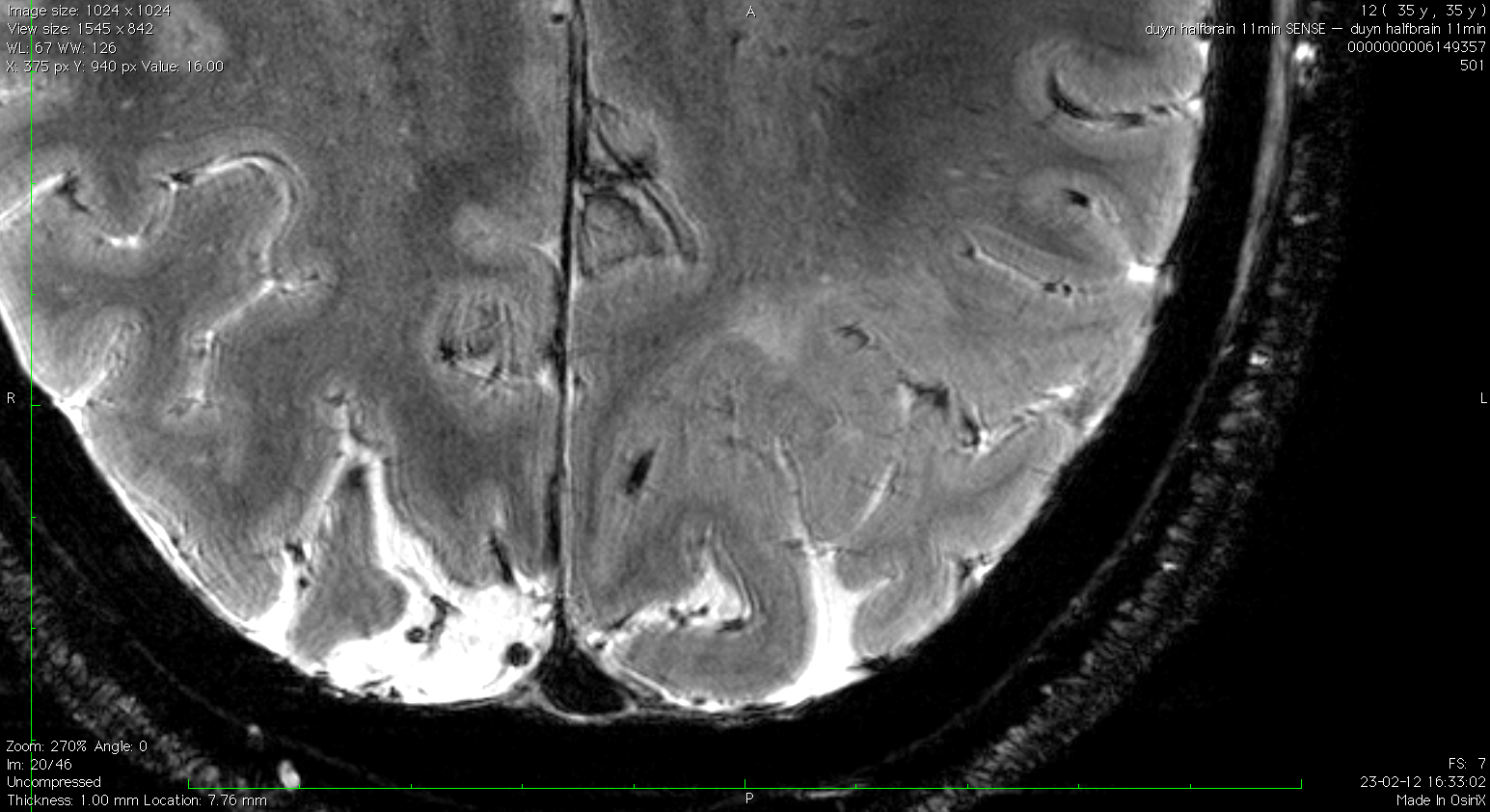


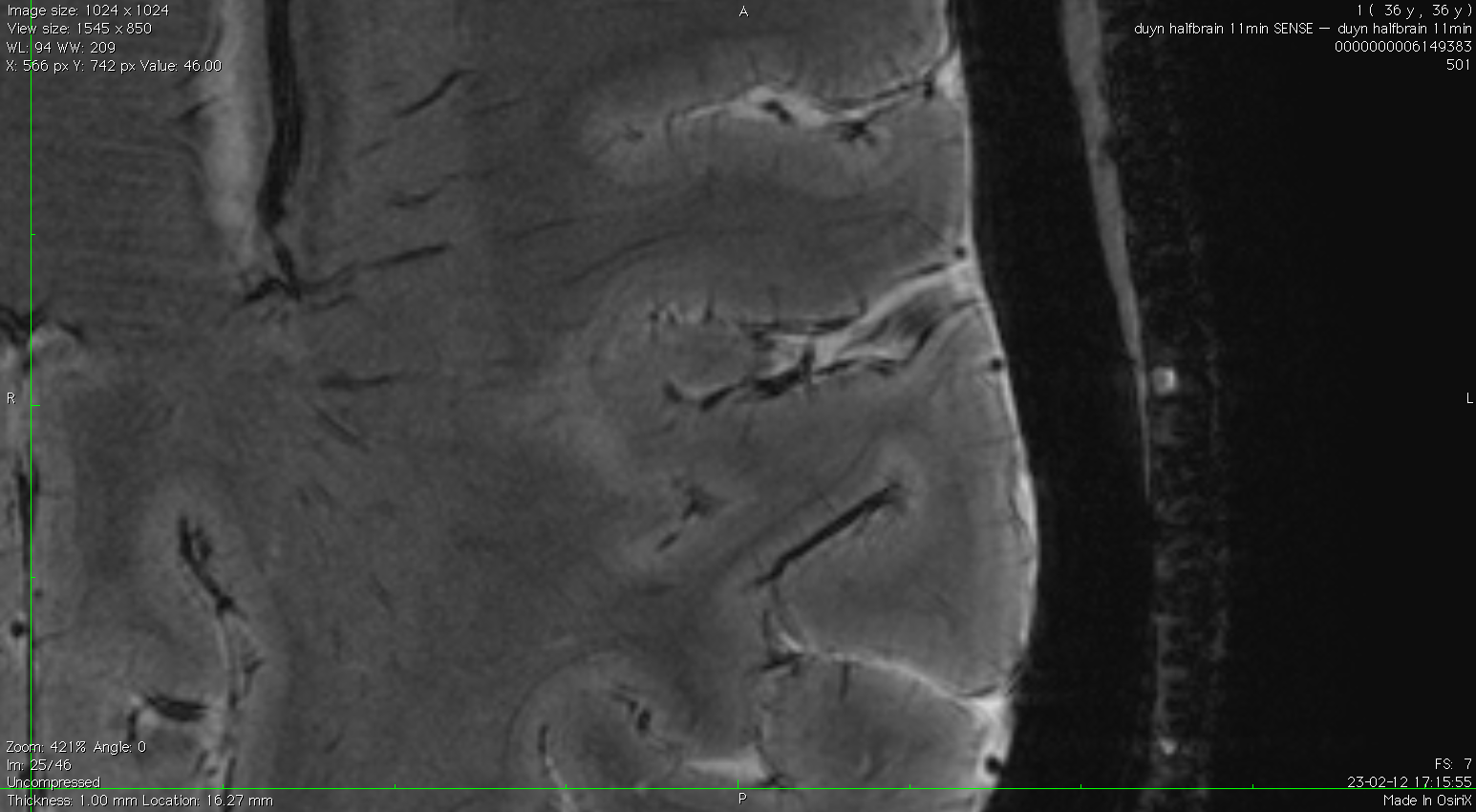

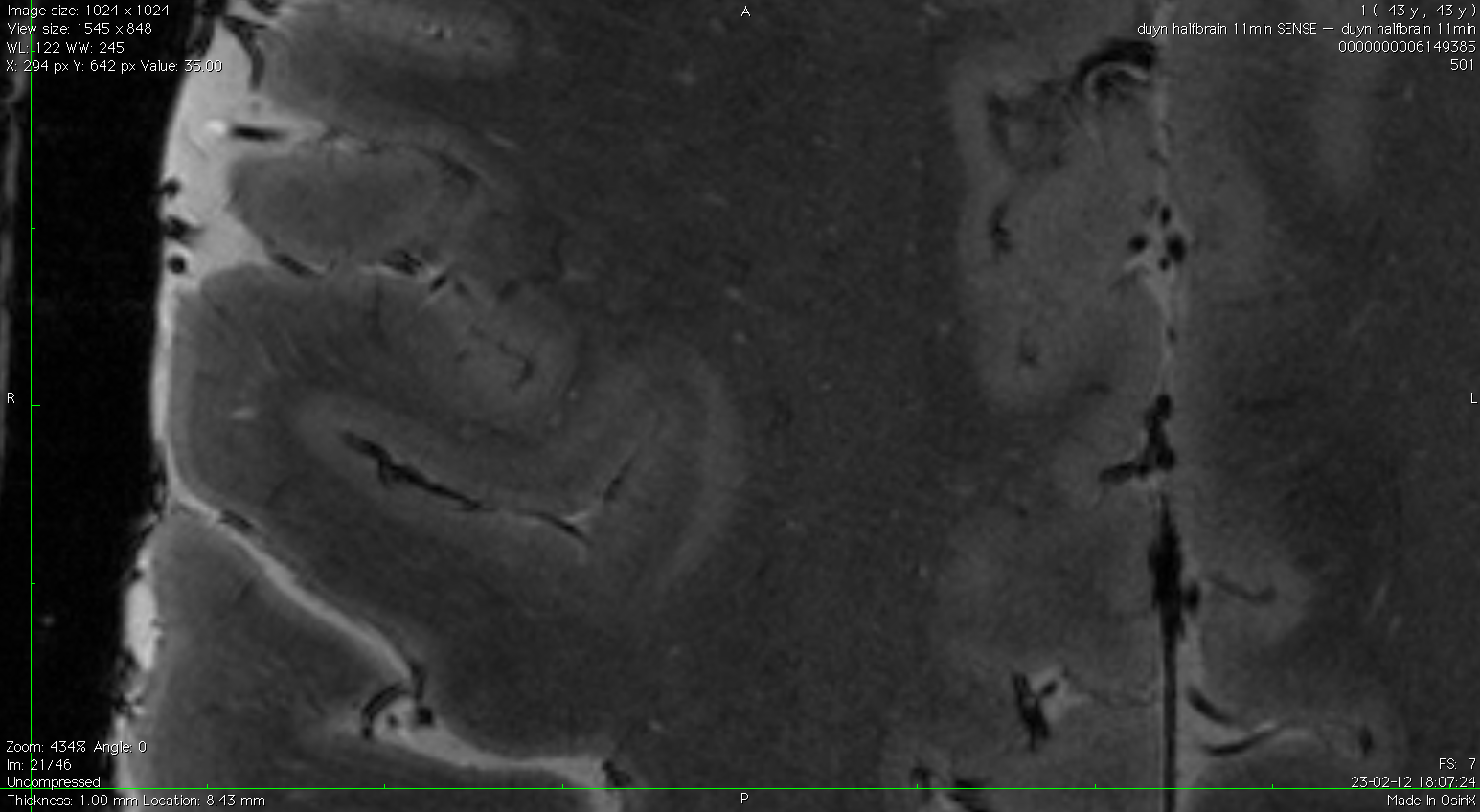


Supplement figure 4: patient 2 to 11: Details of T2* images of FCD on 7T.

Supplement: Supplementary file 2 — Supplementary material 2 (DOCX 4995 kb) [file 13760_2016_662_MOESM2_ESM.docx]

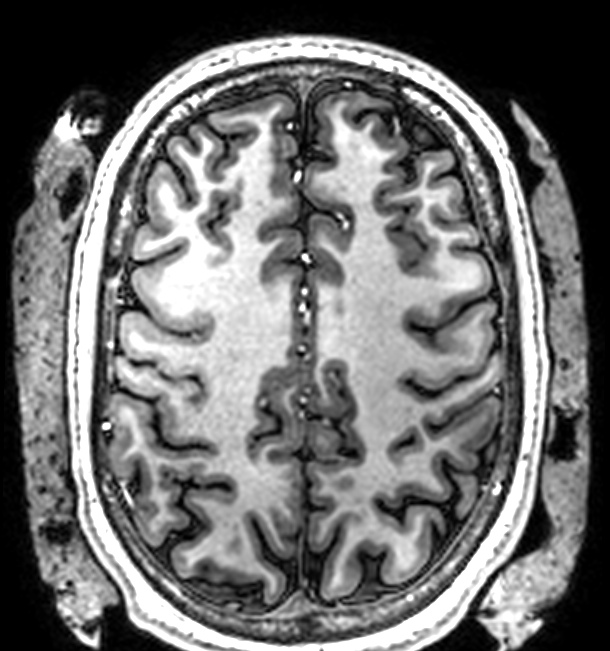

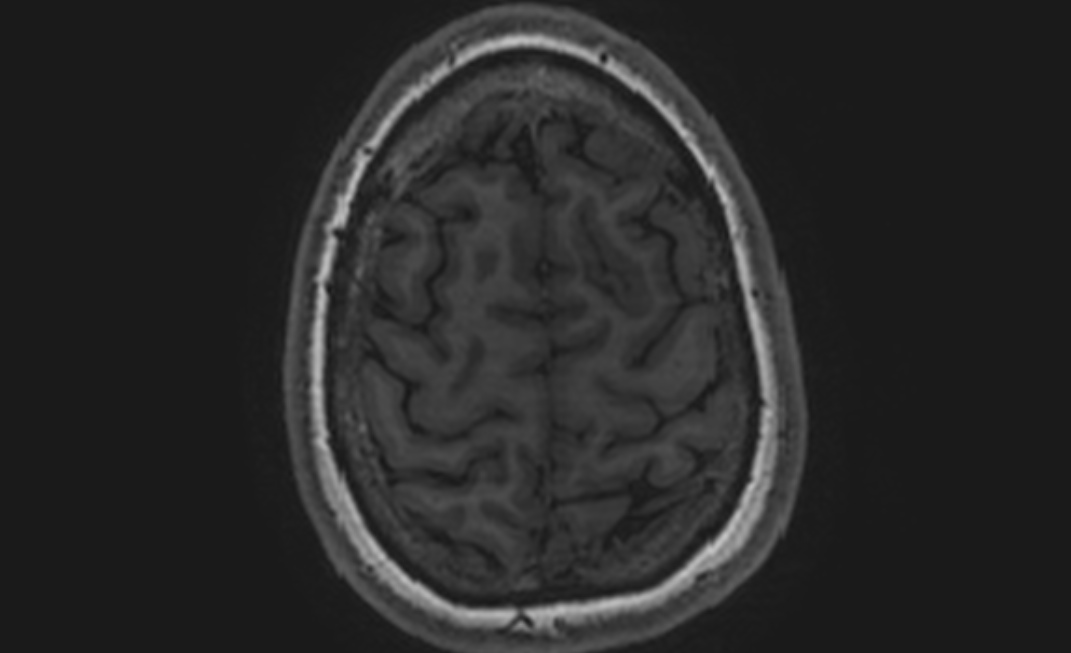


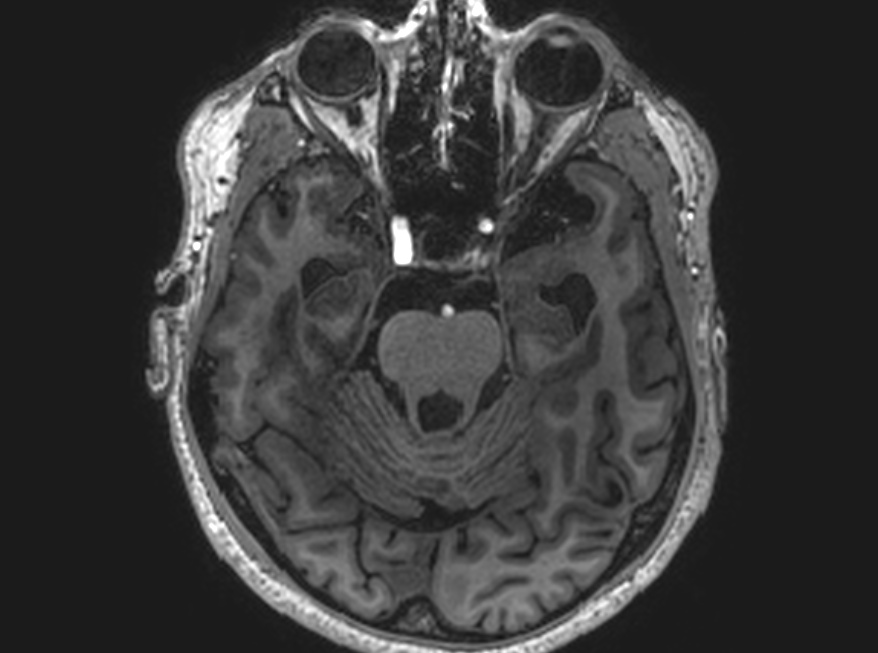

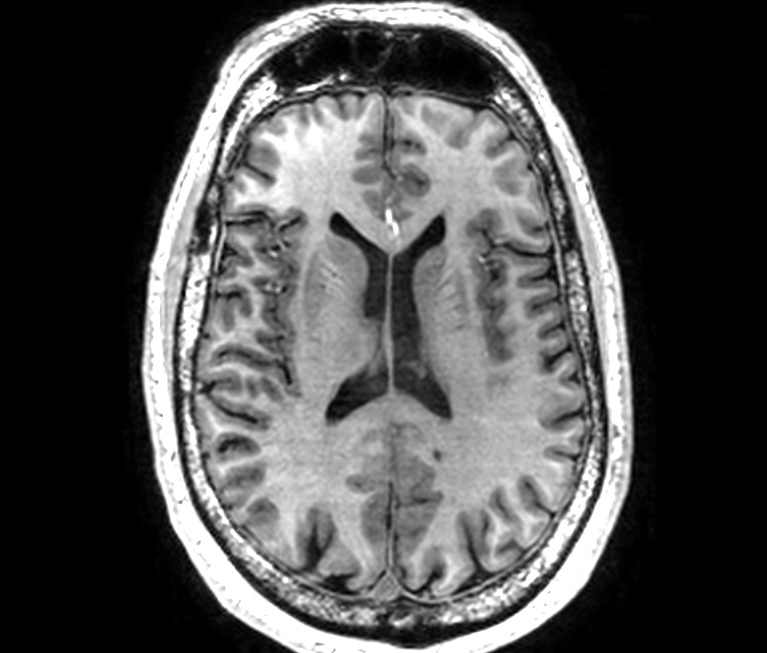


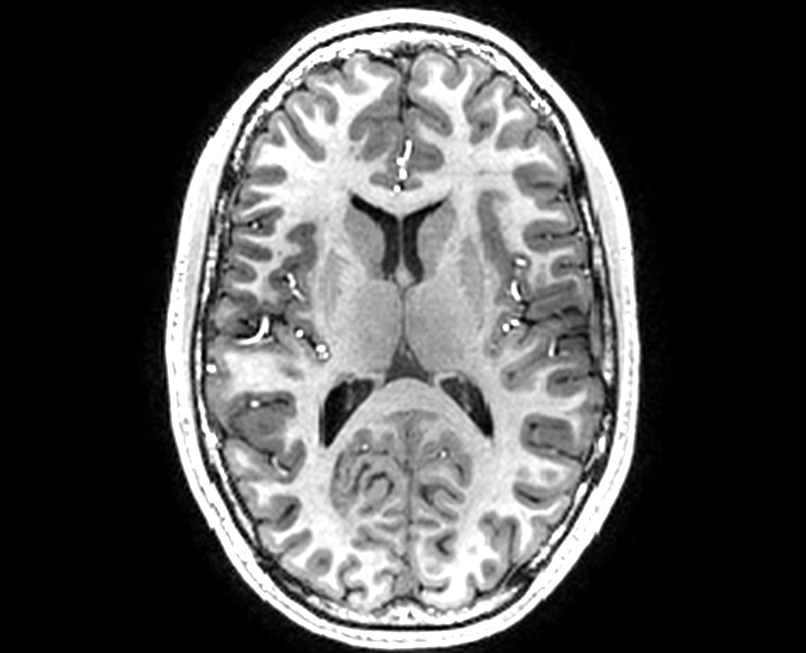

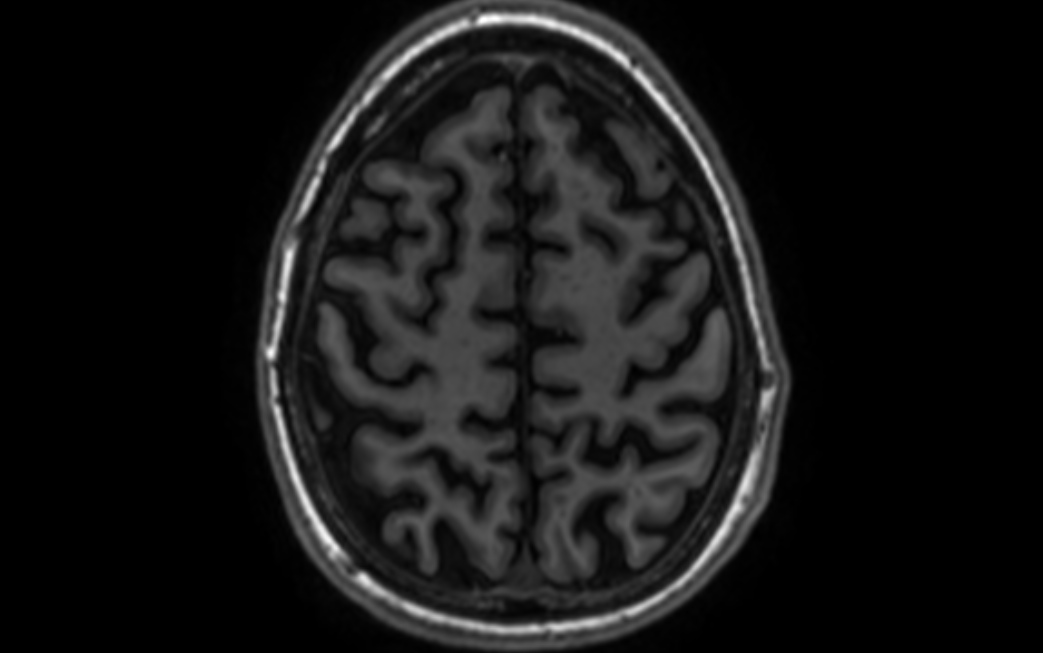


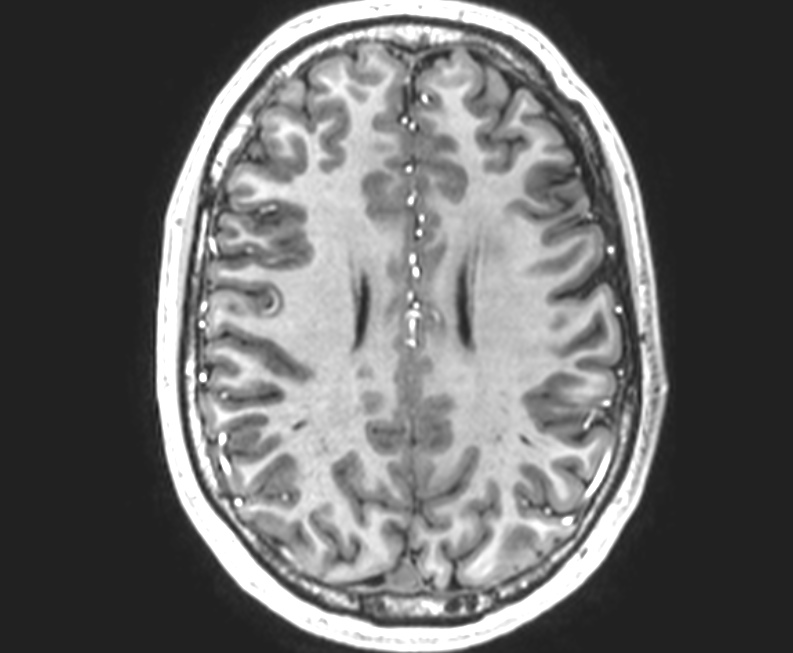

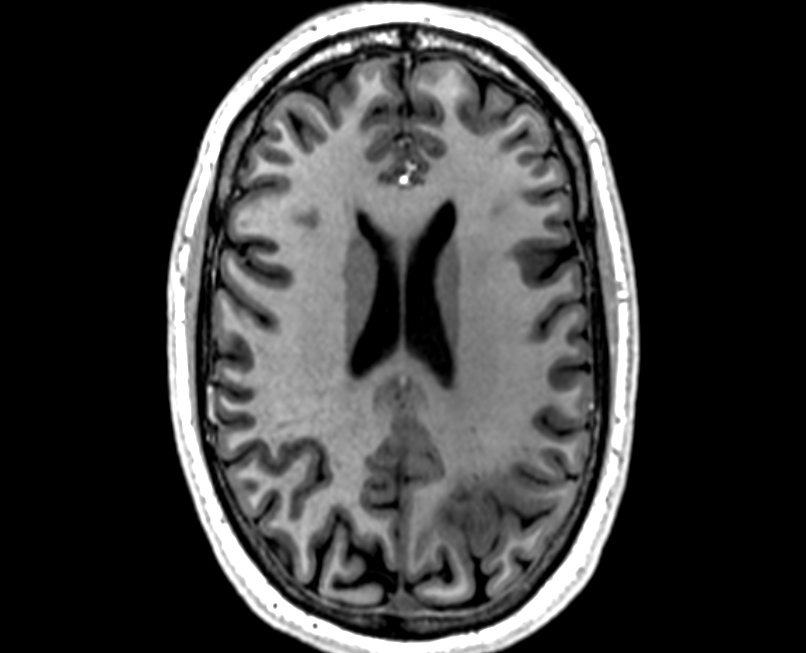


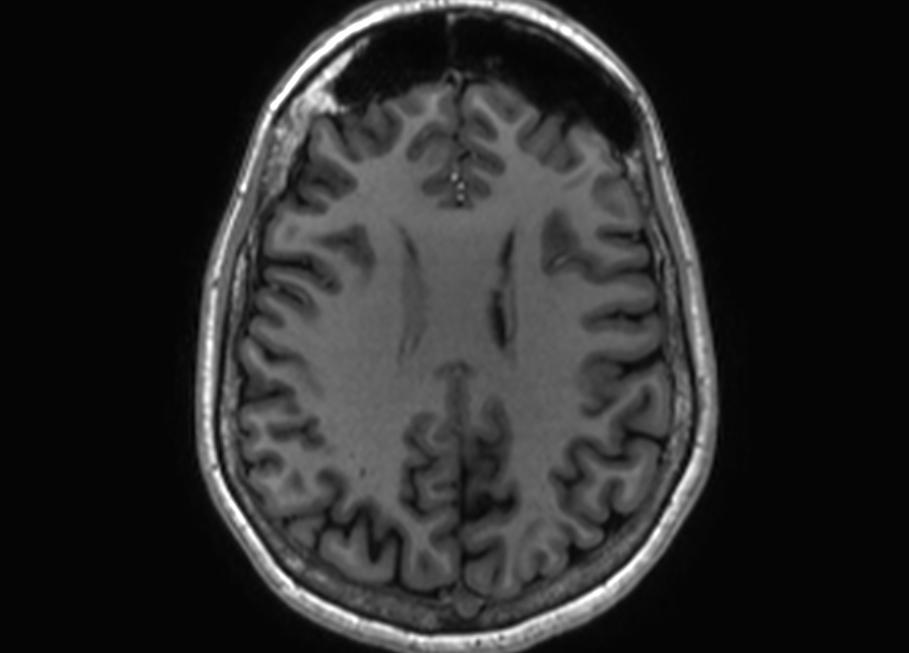

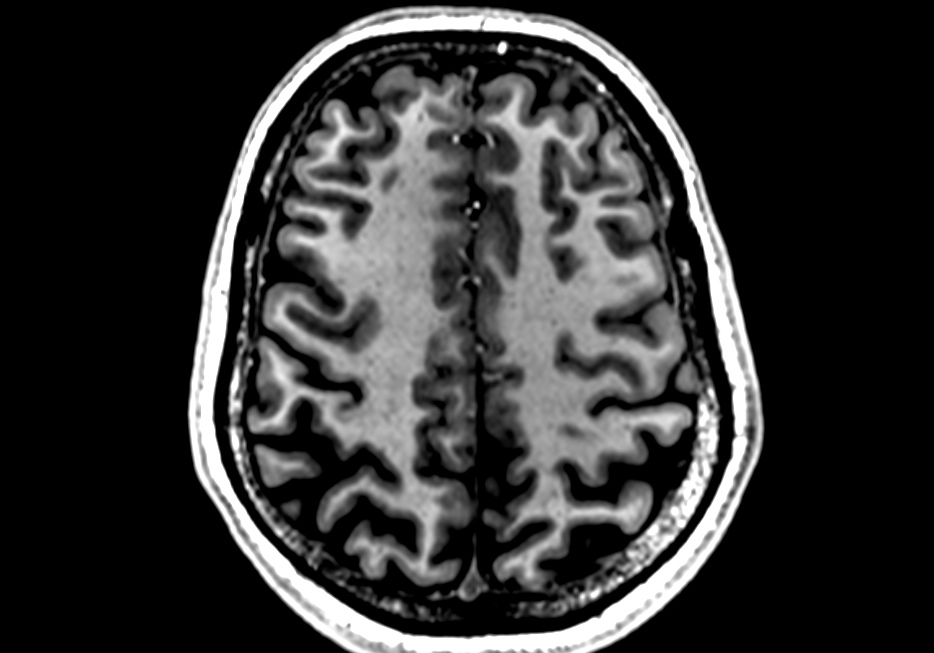


Supplement figure 1: patient 2 to 11, T1 transversal.

Supplement: Supplementary file 3 — Supplementary material 3 (DOCX 951 kb) [file 13760_2016_662_MOESM3_ESM.docx]

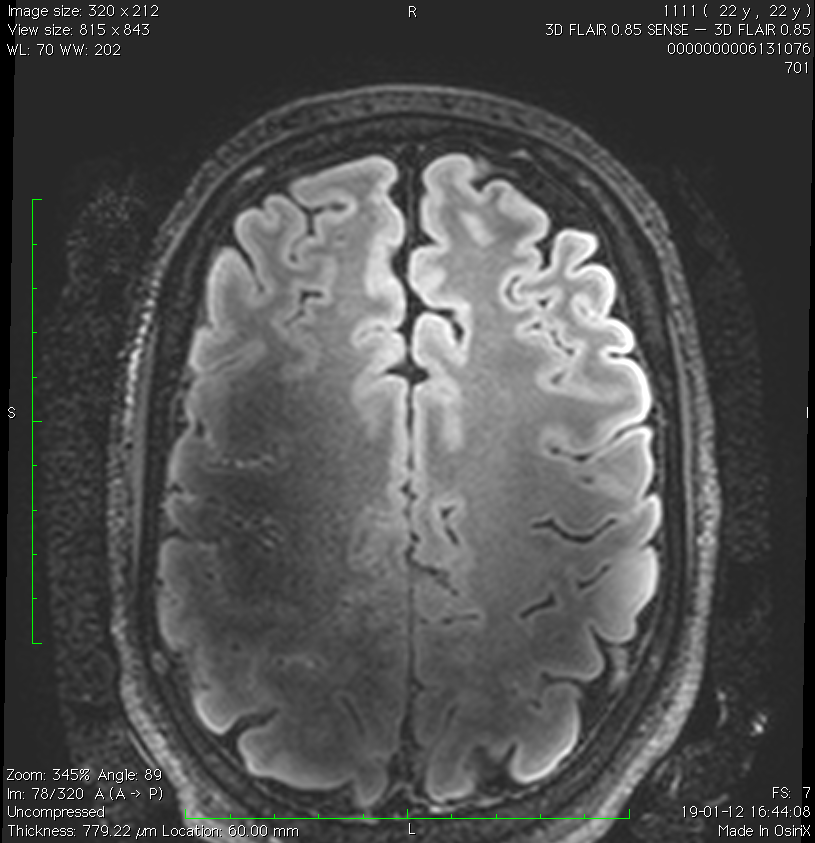

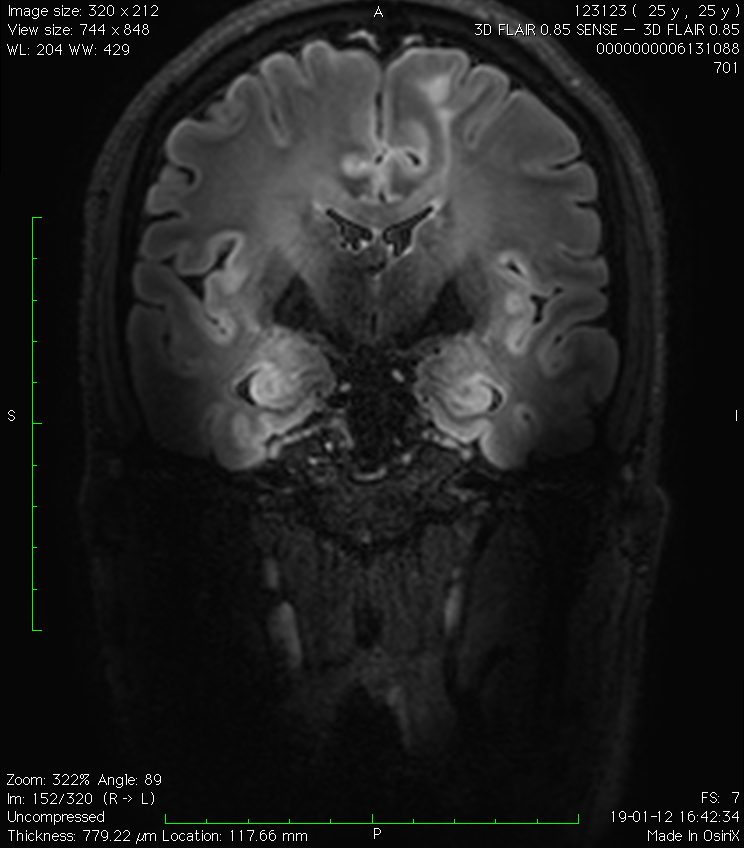


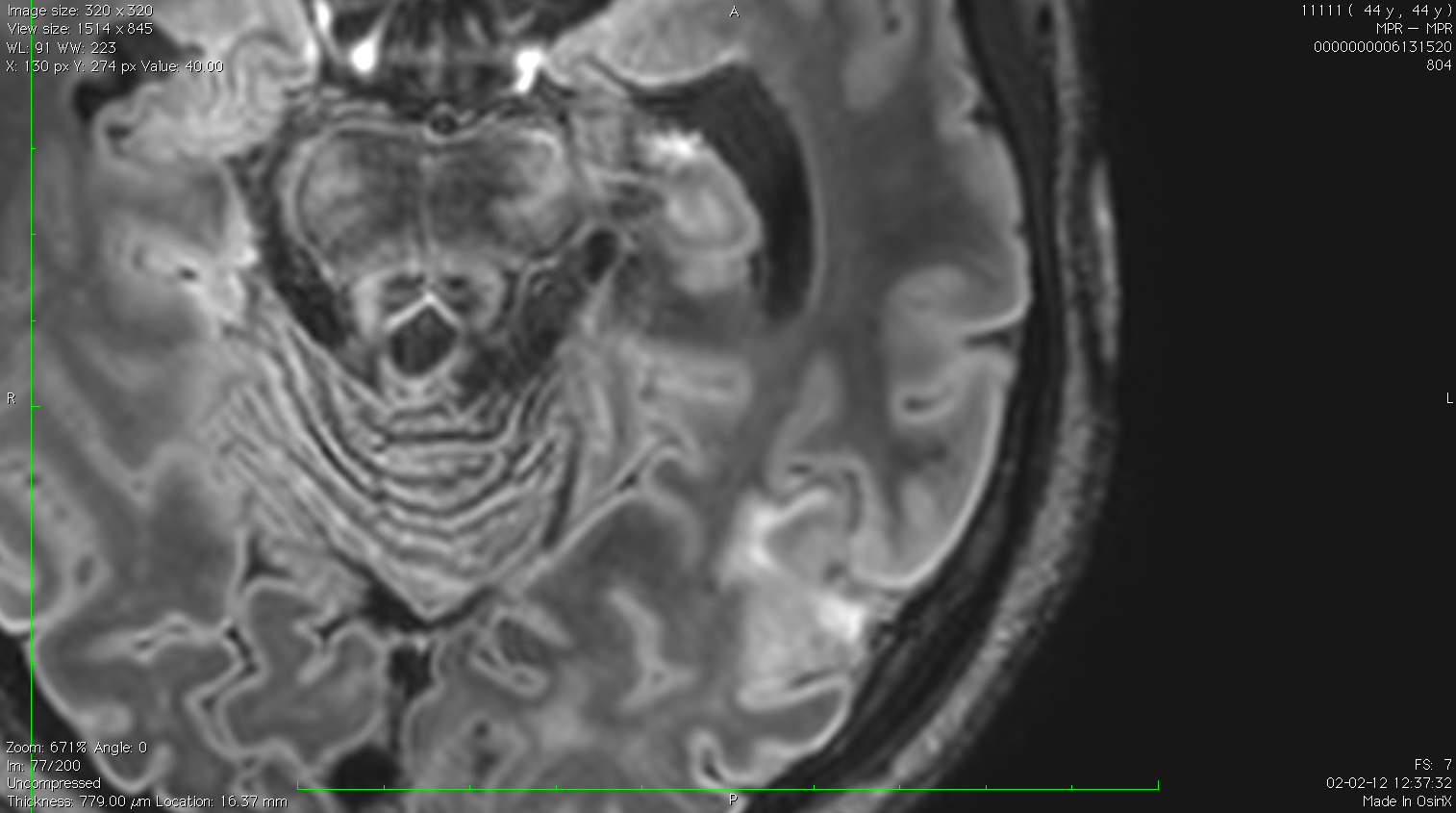

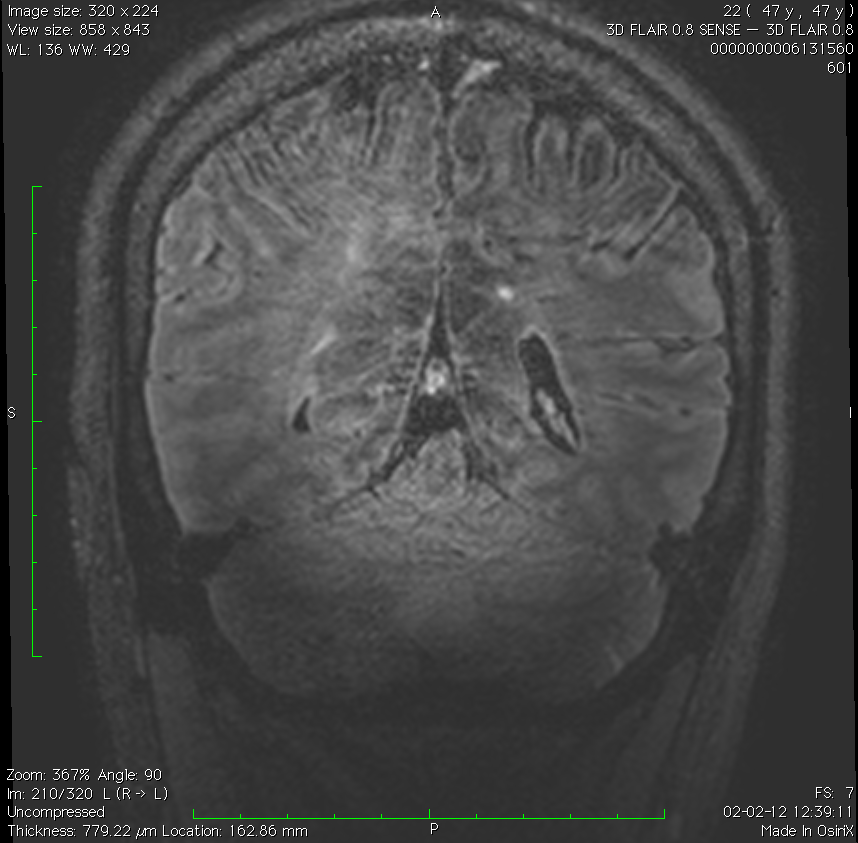


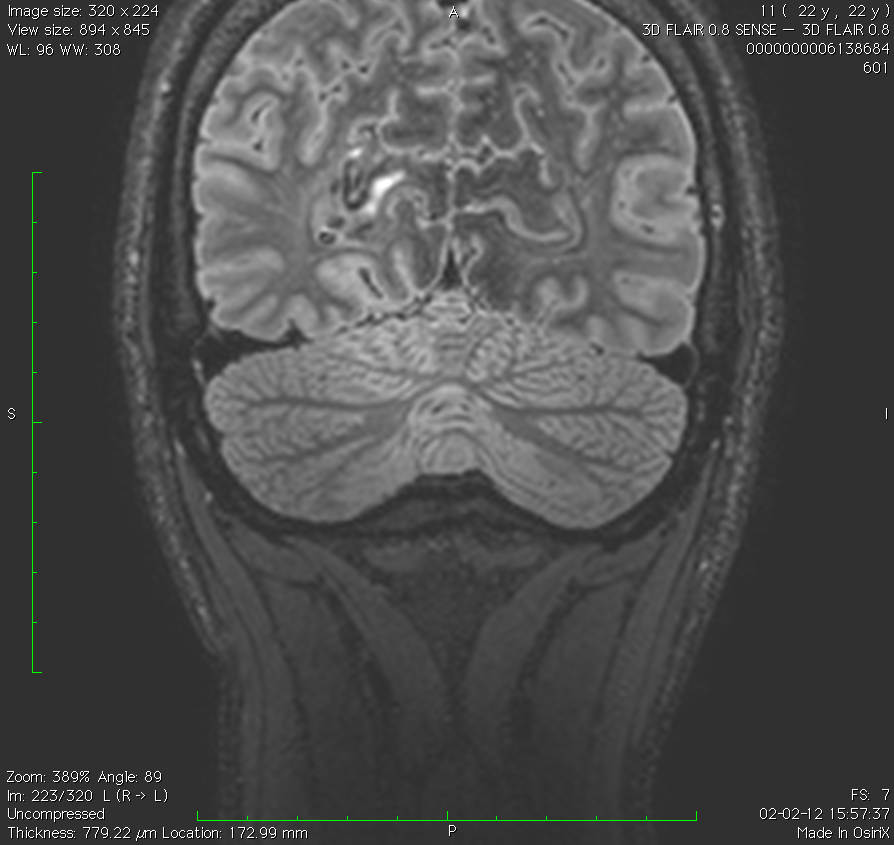

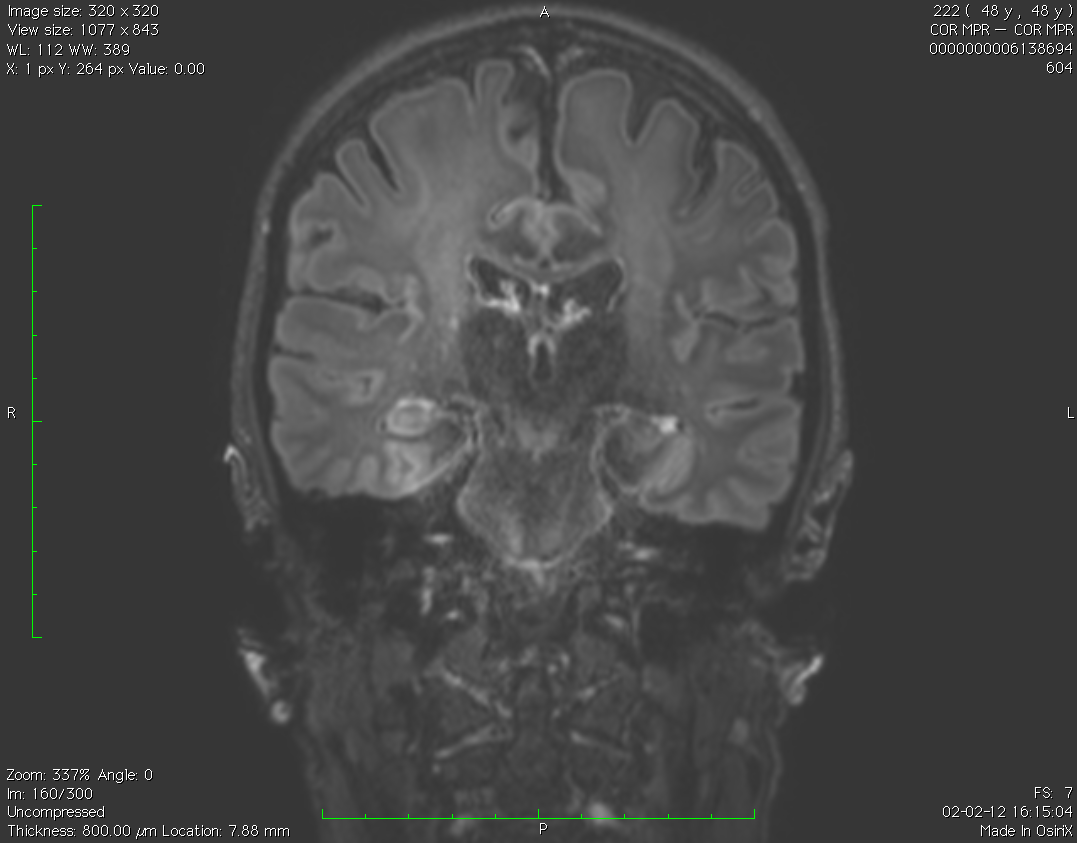


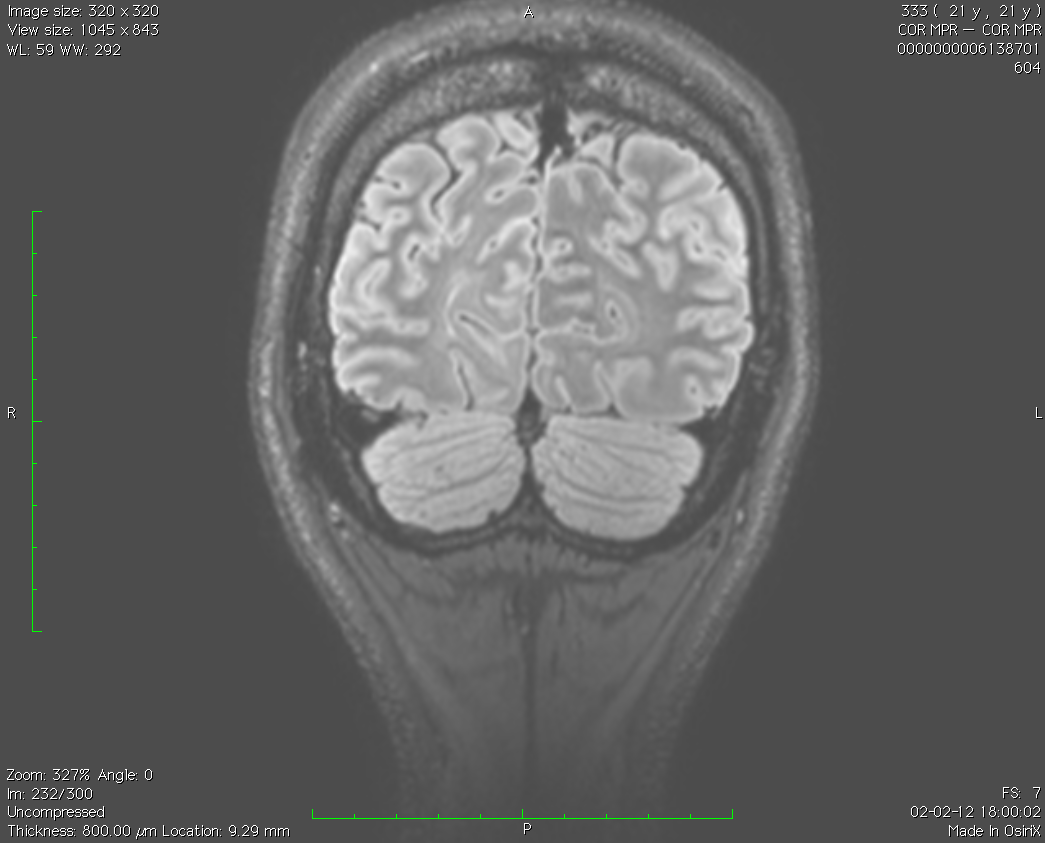

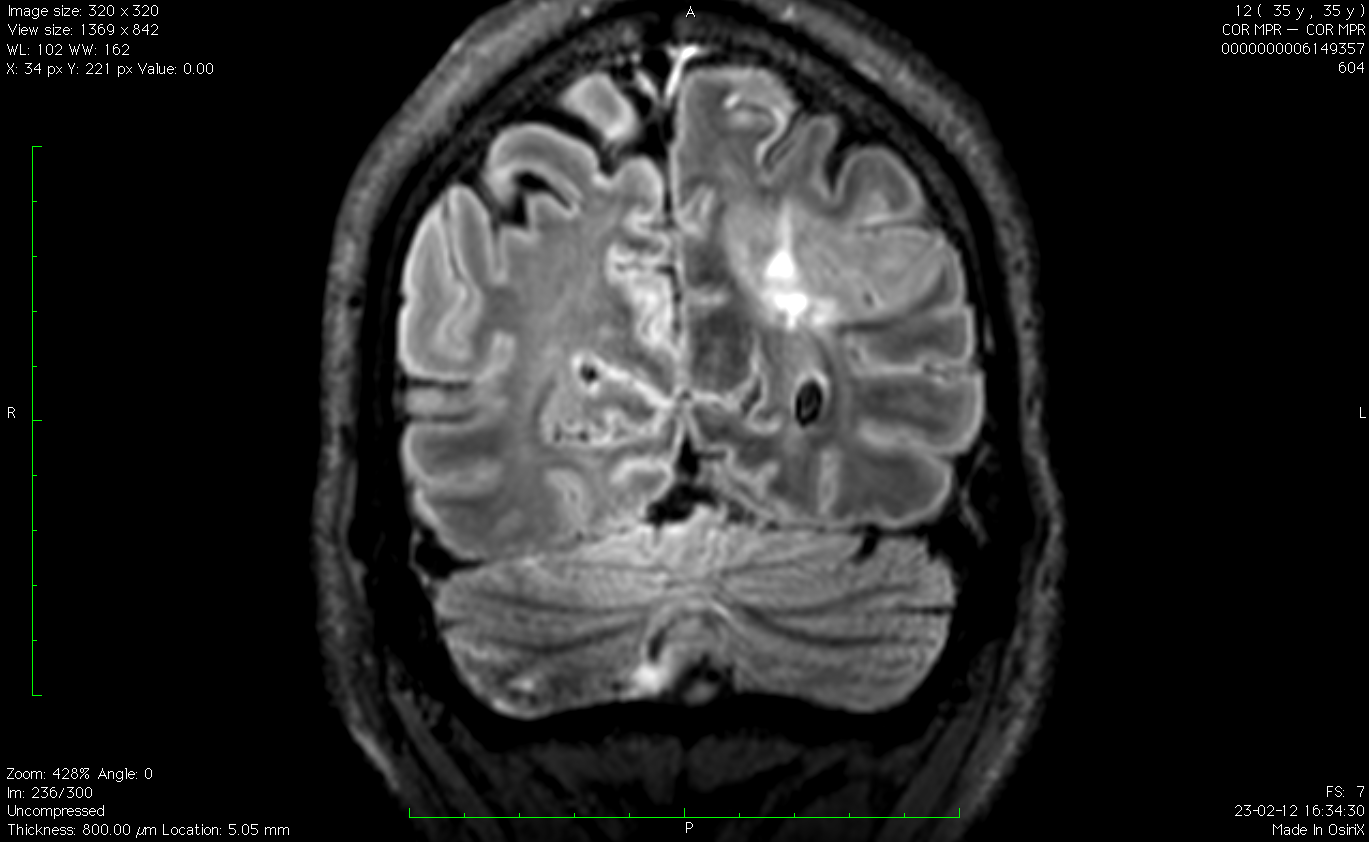


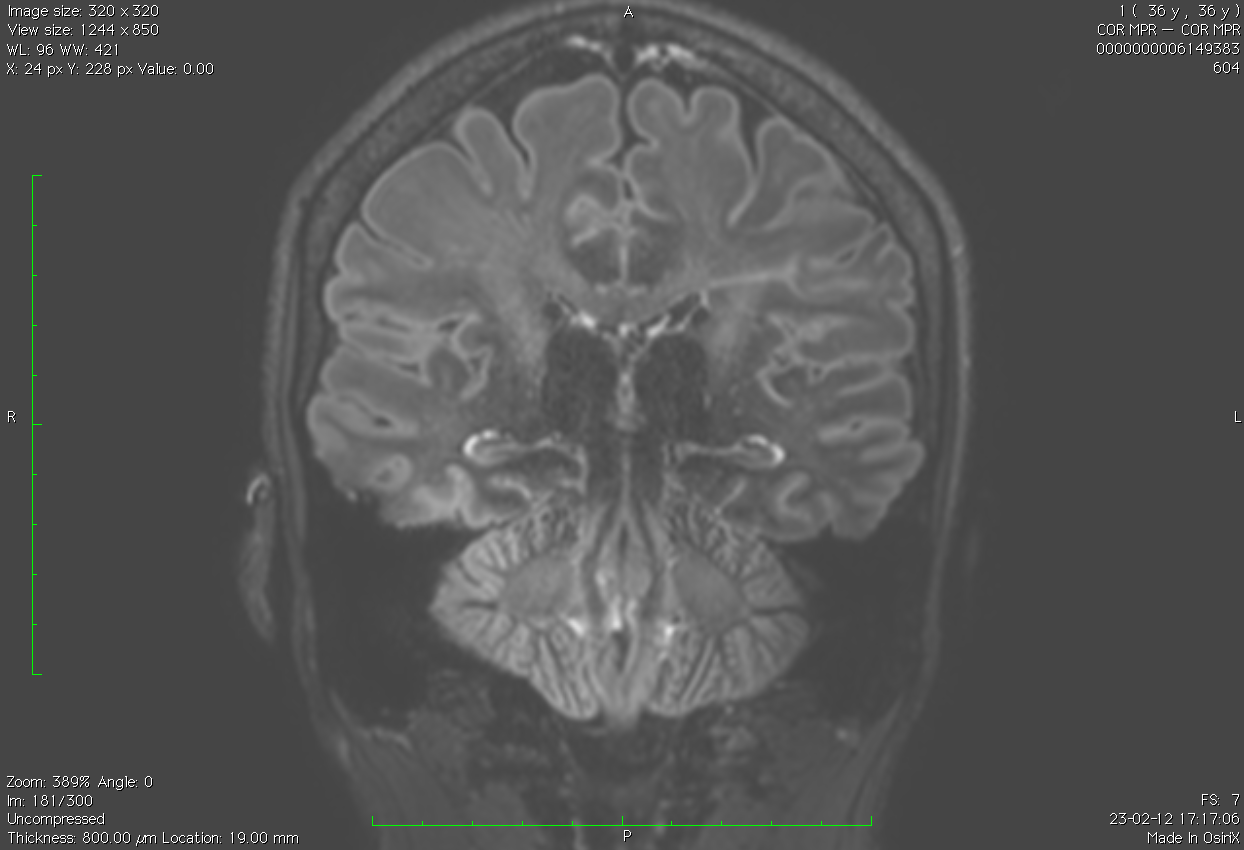

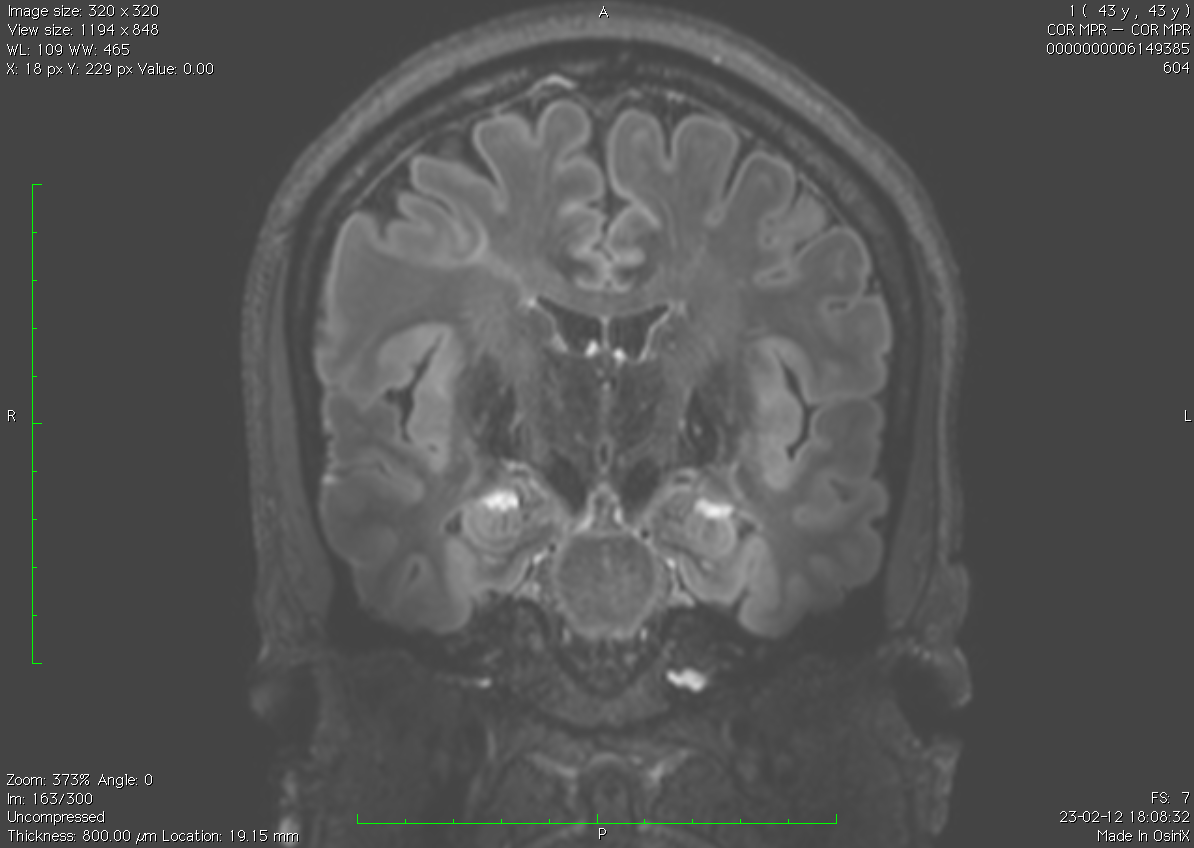


Supplement figure 3: patient 2 to 11: FLAIR images of FCD on 7T.

Supplement: Supplementary file 4 — Supplementary material 4 (DOCX 2749 kb) [file 13760_2016_662_MOESM4_ESM.docx]
